# A densely modified M^2+^-independent DNAzyme that cleaves RNA efficiently with multiple catalytic turnover†
†Electronic supplementary information (ESI) available. See DOI: 10.1039/c7sc04491g


**DOI:** 10.1039/c7sc04491g

**Published:** 2018-01-16

**Authors:** Yajun Wang, Erkai Liu, Curtis H. Lam, David M. Perrin

**Affiliations:** a Chemistry Dept. , UBC , 2036 Main Mall , Vancouver , BC V6T1Z1 , Canada . Email: dperrin@chem.ubc.ca

## Abstract

Sequence-specific cleavage of RNA targets in the absence of a divalent metal cation (M^2+^) has been a long-standing goal in bioorganic chemistry. Herein, we report the *in vitro* selection of novel RNA cleaving DNAzymes that are selected using 8-histaminyl-deoxyadenosine (dA^im^TP), 5-guanidinoallyl-deoxyuridine (dU^ga^TP), and 5-aminoallyl-deoxycytidine (dC^aa^TP) along with dGTP. These modified dNTPs provide key functionalities reminiscent of the active sites of ribonucleases, notably RNase A. Of several such M^2+^-free DNAymes, DNAzyme 7-38-32 cleaves a 19 nt all-RNA substrate with multiple-turnover, under simulated physiological conditions wherein only 0.5 mM Mg^2+^ was present, attaining values of *k*_cat_ of 1.06 min^–1^ and a *K*_M_ of 1.37 μM corresponding to a catalytic efficiency of ∼10^6^ M^–1^ min^–1^. Therefore, Dz7-38-32 represents a promising candidate towards the development of therapeutically efficient DNAzymes.

## Abstract 2

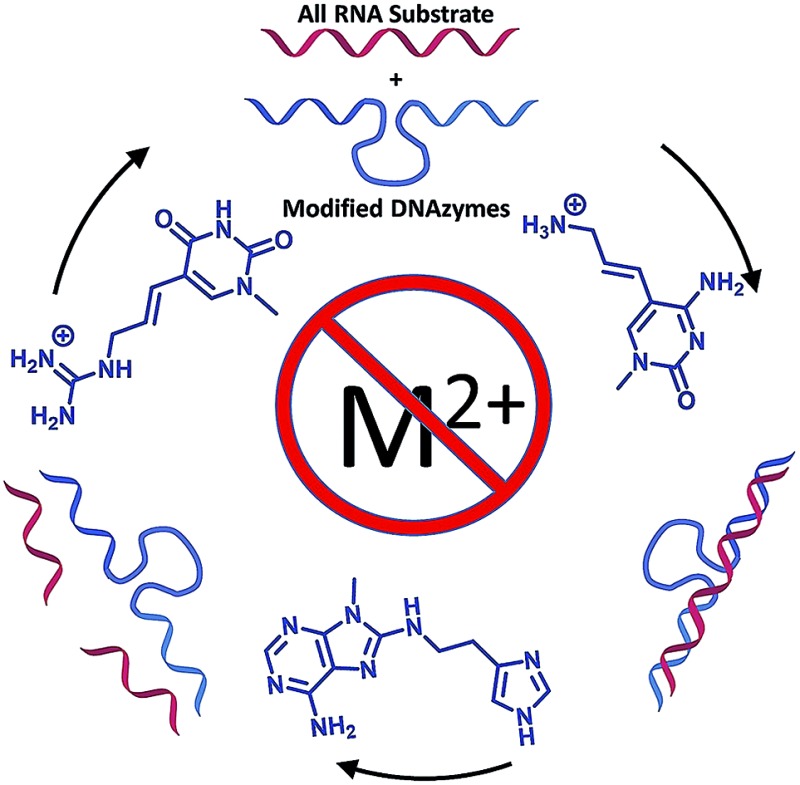
Modified dNTPs permit selection of DNAzymes that cleave RNA targets in the absence of a divalent metal cation (M^2+^) to meet a long-standing goal in bioorganic chemistry.

## Introduction

For over three decades, the development of catalysts capable of sequence-specific RNA cleavage has represented a formidable intellectual challenge with ramifications for both artificial enzyme design and the therapeutic destruction of pathogenic mRNAs. Two general lines of investigation have captured the imagination of chemists and biologists: the first being the rational design of catalysts that mimic ribonucleases, the second being the re-engineering ribozymes and selection of deoxyribozymes (DNAzymes). Interest in these catalysts endures because multiple catalytic turnover affords potential therapeutic advantages for using a sub-stoichiometric amount of drug with respect to mRNA target, the destruction of which can potentially eliminate hundreds of disease-related protein molecules.

The rational design of RNA-cleaving catalysts has relied on synthetic organic chemistry to conjugate imidazoles, cationic amines, and cationic guanidines to various scaffolds.^1^–^10^ These functionalities are chosen because they are found at the active sites of various M^2+^-independent ribonucleases (*e.g.* RNase A),^11^–^13^ where they play key roles of general acid/base catalysis and electrostatic stabilization. To ensure sequence-specificity, such RNase A mimics have been conjugated to oligonucleotides,^14^–^19^ PNAs^20^–^22^ or peptides^23^ that are used to deliver elements of sequence specificity. These approaches, as reviewed,^24^,^25^ continue to address challenges in terms of ensuring ordered binding, high rates, high specificity, and multiple turnover.

The discovery of self-splicing ribozymes opened new paradigms in biocatalysis that led to re-engineered ribozymes capable of classical Michaelis–Menten kinetics: saturable substrate binding, product release, and most importantly, multiple catalytic turnover.^26^,^27^ Although the hammerhead and hairpin ribozymes show strong M^2+^-dependence for optimal catalytic activity,^28^ several studies suggested a M^2+^-independent mechanism whereby Mg^2+^ cations play the ancillary role of supporting a catalytically competent fold wherein p*K*_a_-perturbed nucleobases play direct catalytic roles of acid–base catalysis.^29^–^34^ Nevertheless, the need for relatively high Mg^2+^ concentrations to achieve catalytic competence, along with a general requirement for a free 2′-OH for full catalytic activity, and the marked serum-instability of RNA due to the 2′-OH, diminished the prospects of therapeutic ribozymes.

Combinatorial *in vitro* selection technologies^35^–^38^ enabled the discovery of RNA-cleaving DNAzymes,^39^,^40^ which became more attractive as potential therapeutics due to the relative stability of DNA compared to RNA. Only a few DNAzymes have been selected to cleave all-RNA targets,^41^,^42^ two notable examples of which are DNAzyme 8-17 and DNAzyme 10-23 (Dz10-23). Dz10-23 is a Mg^2+^-dependent RNA-cleaving DNAzyme,^43^,^44^ which achieves near-catalytic perfection (*k*_cat_/*K*_M_ ∼ 10^9^ M^–1^ min^–1^) at 10 mM Mg^2+^.^45^,^46^ One carefully designed study provided indirect evidence of intracellular catalysis: a catalytically active variant of Dz10-23 gave ∼20% reduction in protein expression while a catalytically inactive variant gave *ca.* ∼10% reduction in protein expression (mRNA cleavage was not ascertained, only luciferase expression). Notably, a variant of Dz10-23 with 2′OMe nucleosides in the catalytic core gave ∼40% reduction in luciferase expression while the corresponding inactive mutant gave no reduction.^47^ Paralleling this work, other DNAzymes based on Dz10-23 have entered phase-I/II clinical trials.^48^,^49^

Despite these promising results, only a few reports provide direct evidence of mRNA cleavage in cells.^50^,^51^ Notably, first- and second-order rate constants fall 2–4 orders of magnitude under simulated physiological conditions (0.5 mM Mg^2+^). One study showed that at 0.5 mM Mg^2+^, Dz10-23 collapses into a catalytically inactive conformation raising questions as to the basis for mRNA silencing in cells.^45^ Use of a photoactivatable Dz10-23 showed that the observed mRNA-silencing activity in cells is primarily due to antisense effects, not catalysis.^46^ Taken together, these studies corroborate a long-standing hypothesis that the intracellular free M^2+^ concentration (Mg^2+^ < 0.5 mM; Ca^2+^, Zn^2+^, and Cu^2+^ < 50 nM)^52^–^54^ is simply too low to support efficient multiple turnover of RNA cleavage by unmodified M^2+^-dependent nucleic acid catalysts.

Recognizing the value in circumventing the need for Mg^2+^, several labs selected Mg^2+^-independent RNA-cleaving DNAzymes at pH ∼ 7 in the presence of 0.25–1 M monovalent cations (Na^+^, K^+^). In two cases, catalytic DNA sequences showed low rate constants for self-cleavage (*k*_obs_ ∼ 10^–3^ min^–1^) and were incapable of multiple turnover,^55^,^56^ whereas a third such DNAzyme, with similar rate constants, showed two turnovers in 100 h.^57^ Recently, a M^2+^-independent Na^+^-dependent DNAzyme selected to cleave at a single ribophosphodiester linkage showed multiple catalytic turnover with an apparent *k*_cat_ of 0.026 min^–1^ in 50 mM NaCl, at 19–21 °C.^58^ Yet cleavage of an all-RNA target under similar conditions is currently unknown.

Despite considerable work towards sequence-specific RNA cleavage for potential therapeutic applications, multiple turnover catalysis under the low Mg^2+^ regime within cells is rarely observed. Nevertheless, the enormous promise inspired by ribozymes, DNAzymes, and synthetic RNase A mimics is not diminished; rather, work from the past three decades presents a heightened impetus to identify M^2+^-independent catalysts.

One elegant approach has been to introduce nucleosides modified with imidazoles or amines into the catalytic cores of Dz10-23 and Dz8-17 and test for activity at low concentrations of Mg^2+^.^59^–^61^ An alternative approach uses deoxyribonucleoside triphosphates (dXTPs) that are covalently modified with imidazoles and cationic amines,^62^–^64^ which are used in combinatorial DNAzyme selection to discover DNAzymes that function as sequence-specific RNase A mimics.^65^,^66^ In such selections, the modified nucleoside triphosphates must replace unmodified counterparts in enzyme-mediated DNAzyme synthesis and should be expected to enhance selected activity over unmodified congeners.

**Fig. 1:**
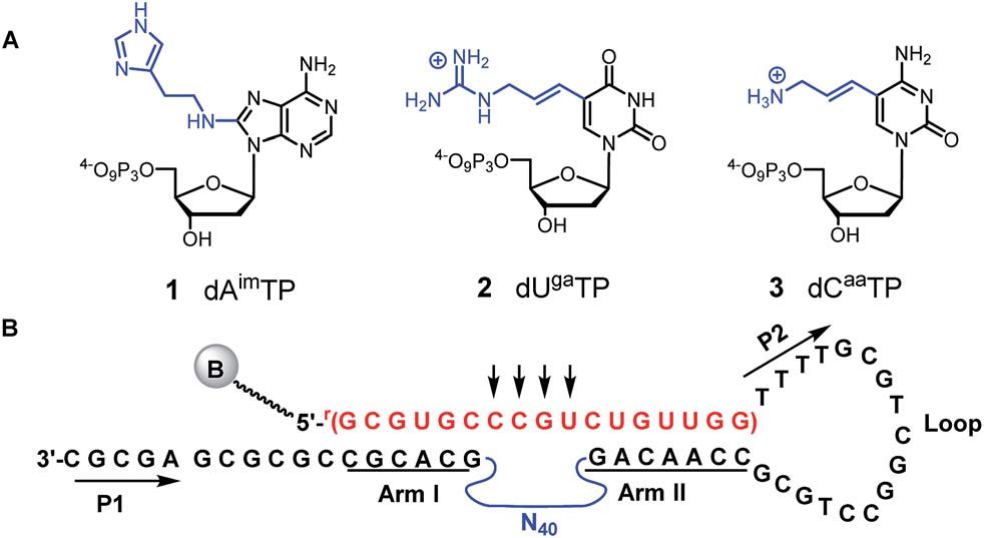
(A) Chemical structures of modified deoxynucleotides for RNase A mimic: dA^im^TP 1, dU^ga^TP 2, dC^aa^TP 3. (B) Unimolecular construct for *in vitro* selection of all-RNA cleaving DNAzymes. The 17 nt all-RNA substrate is depicted in red and is taken from the HIV-LTR untranslated message; the potential cleavage was directed at either of the four unpaired RNA linkages (indicated by arrows) opposite the initial random region (N40); the two fixed sequence regions (P1 and P2) flanking the N40 random region ensuring PCR amplification are depicted in black, with the nucleosides in the substrate recognition arms (arm I and arm II) underlined.

Following our early work on a DNAzyme with imidazoles and amines, which we showed acted as an RNase A mimic on a substrate containing a single ribophosphodiester linkage,^67^,^68^ we used three modified dNTPs: 8-histaminyl-dATP (dA^im^TP), 5-aminoallyl-dCTP (dC^aa^TP), and 5-guanidinoallyl-dUTP (dU^ga^TP) (**1–3**, Fig. 1A) to discover highly active, M^2+^-independent DNAzymes, which showed significantly increased *k*_cat_ values compared to unmodified catalysts, and in particular, the importance of the guanidinium cation for considerable thermostability.^69^–^71^

Nevertheless, to date, these DNAzymes cleave only chimeric substrates containing a single ribophosphodiester linkage and thus would never function as sequence-specific RNase A mimics for all-RNA substrates. Inspired by the considerable history in this field, herein we describe the discovery a M^2+^-independent DNAzyme that efficiently cleaves an all-RNA target with multiple catalytic turnover by virtue of these three protein-like functionalities.

## Results

### *In vitro* selection

To select for all-RNA cleavage, we introduced a 17 nt all-RNA target, derived from the HIV-1 LTR promoter, into a self-cleaving construct that was outfitted with a large loop that was designed to reduce secondary structure and to ease the conversion of a *cis*-acting DNAzyme to a *trans*-acting one. The large loop was anticipated to separate the guide arms and incipient catalytic motif from the target to favour a pseudo-intermolecular interaction (Fig. 1B) and may have contributed to success in terms of eventually selecting catalysts that could function with multiple turnover.

**Fig. 2:**
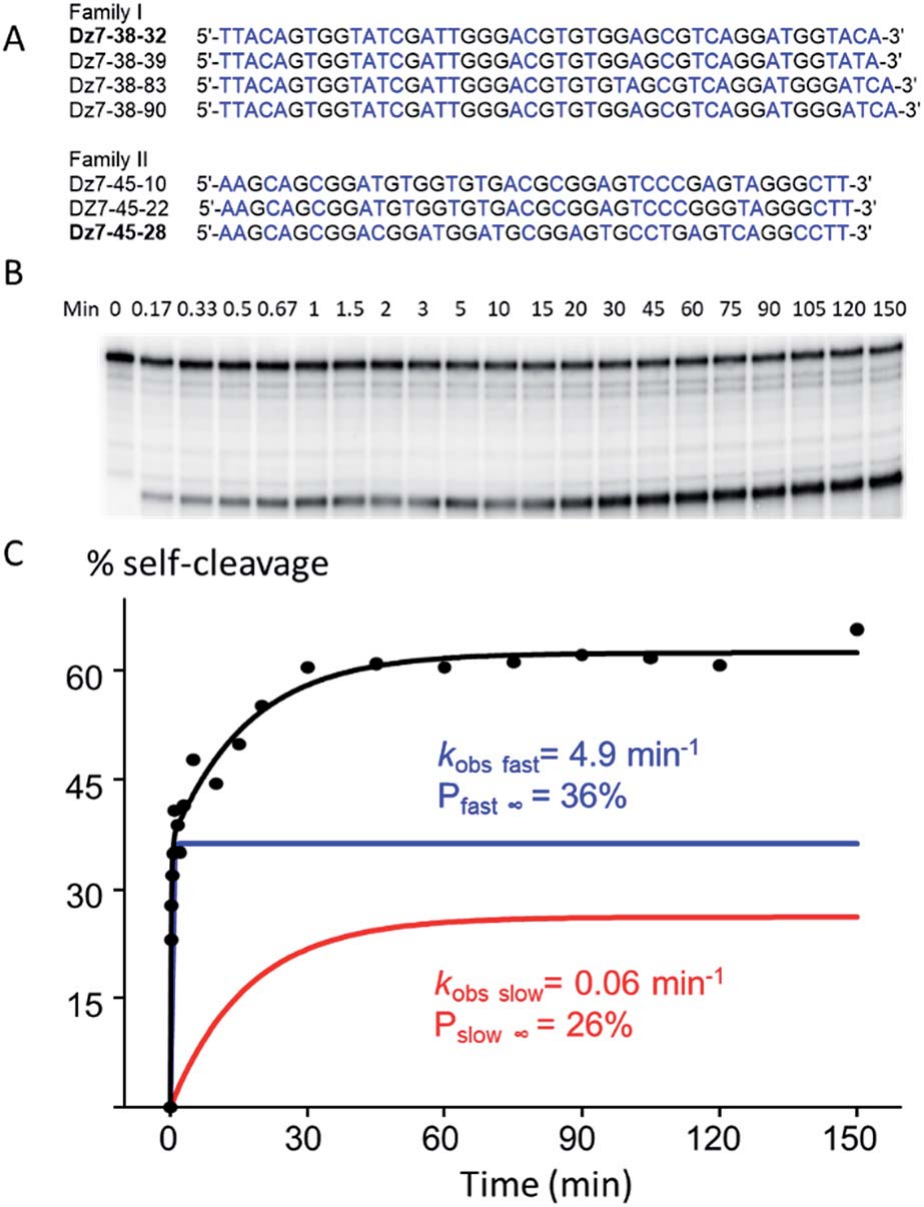
*cis*-Cleavage activity of Dz7-38-32 at 25 °C. (A) Individual sequences of the initially random (N40) region of two families of highly active DNAzymes (T is noted from the sequencing data however is replaced with dU^ga^ in the modified sequences). (B) Autoradiograph of a representative *cis*-cleavage reaction. (C) Biphasic kinetic plot of c*is*-cleaving Dz7-38-32 (*R*^2^ = 0.98).

Initially, seven rounds of selection gave eleven active sequences belonging to two different families (see Table S1, ESI†), each of which showed an observed self-cleavage rate constant (*k*_obs_) of ∼10^–2^ min^–1^. For re-selection, the unmodified DNA templates of Dz7-38 and Dz7-45 were re-synthesized on the solid-phase at ∼15% degeneracy for each position encompassing the catalytic core and re-amplified using mutagenic PCR to provide templates for the production of modified DNAzymes. Fourteen additional selection rounds accompanied by mutagenic PCR converged on two families of DNAzymes (Fig. 2A) with 100-fold higher self-cleavage rate constants (*k*_obs_). The appearance of two families suggested the evolution of two very different catalytic motifs that represent different solutions to the same problem and suggest that the added functionalities provide not only increased chemical functionality but greater structural diversity (see Fig. S1 for selection and re-selection progressions in ESI†).

### Self-cleavage activity (*in cis*)

Two clones, one from each DNAzyme family, Dz7-38-32 and Dz7-48-28, were characterized in terms of *cis*-cleavage under M^2+^-free conditions (50 mM cacodylate, pH 7.45, 200 mM NaCl, 1 mM EDTA, at 25 °C). Dz7-38-32 gave an overall self-cleavage yield of 62%. Cleavage was distinctly biphasic: the amplitude of the fast phase (*P*_fast∞_) was calculated to be 36% with an extraordinarily high apparent rate constant (*k*_obs fast_) of 4.9 min^–1^ while the amplitude for the slow phase (*P*_slow∞_) was calculated to be 26% with an apparent catalytic rate constant (*k*_obs slow_) of 0.06 min^–1^. By contrast, Dz7-45-28 showed a higher overall self-cleavage yield however much lower rate constants: a fast phase amplitude (*P*_fast∞_) was calculated to be 52%, with an apparent catalytic rate constant (*k*_obs fast_) of 0.21 min^–1^ and a slow phase amplitude (*P*_slow∞_) of 48%, with a slow phase apparent catalytic rate constant (*k*_obs slow_) of 0.007 min^–1^. The *cis*-cleavage autoradiograph and biphasic fitted curves for Dz7-38-32 are shown in Fig. 2B, while the *cis*-cleavage rate profiles for Dz7-48-28 as well as other sequences given in Fig. 2A, are shown in Fig. S2, ESI.†

To demonstrate the general necessity of all three modified nucleosides, we used primer extension reactions to resynthesize self-cleaving species wherein we replaced one, two, or three modified dXTPs with an unmodified congener. In all cases, the resulting self-cleaving strands displayed near-total loss of cleavage activity, with less than ∼5% cleavage observed over 840 minutes (Fig. S3, ESI†), demonstrating that at least one of each of the three modified nucleotides plays a crucial role in terms of either structure, catalysis or both.

### Multiple turnover cleavage (*in trans*)

Given the superior rate constants observed for Dz7-38-32 self-cleavage, we engineered this species into a *trans*-acting DNAzyme that would function as a true enzyme, heretofore referred to as Dz7-38-32t. To do this, the large loop connecting the substrate to the catalyst was ablated and a biotinylated template was prepared. The *trans*-acting DNAzyme was then enzymatically synthesized by template-directed primer extension reaction. The resulting heteroduplex was trapped on streptavidin-magnetic particles and the catalyst was removed by brief NaOH treatment and collected in a suitable buffer. The catalyst concentration was determined as previously reported;^70^ briefly, the specific radioactivity of Dz7-38-32t was related to that of the α-^32^P-dGTP used in the primer extension reaction as determined by a standard curve generated by autoradiography and quantified by a phosphorimager (Fig. S4, ESI†).

**Fig. 3:**
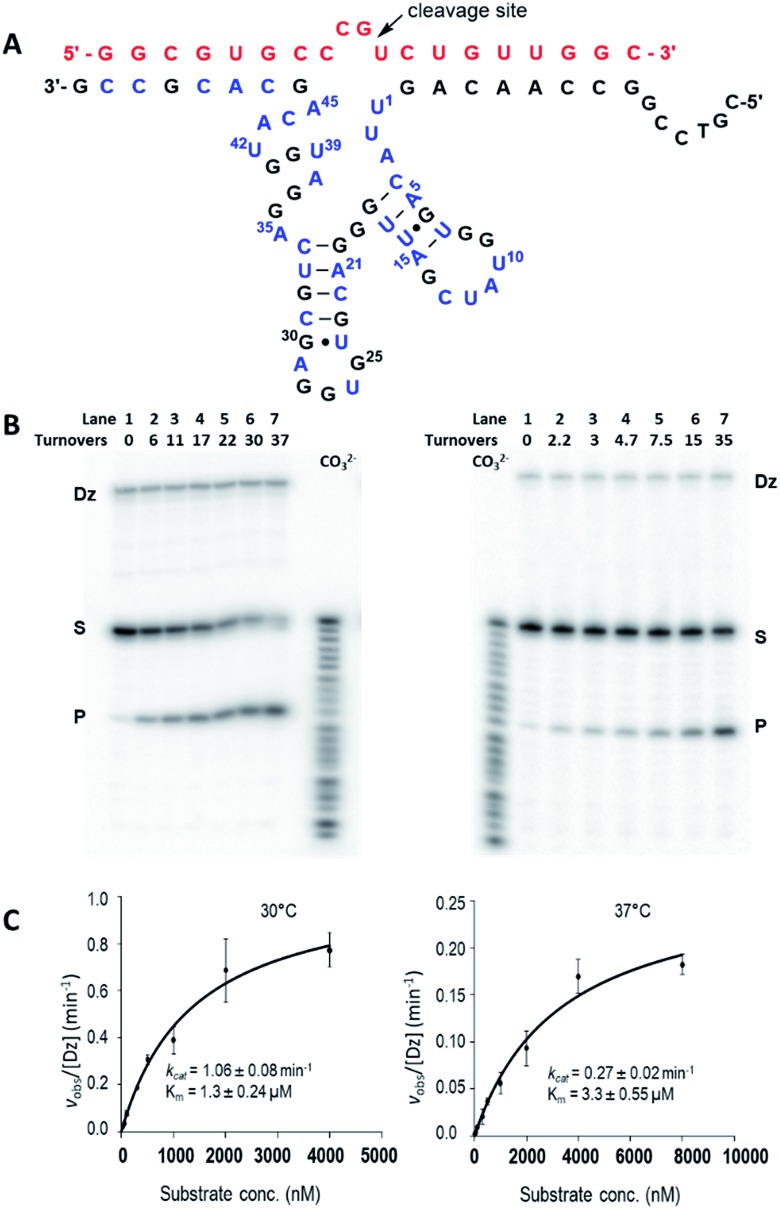
*trans*-Cleavage activity of Dz7-38-32t. (A) Hypothetical 2D structures of Dz7-38-32t (bottom sequence: 3′–5′) in complex with the 19 nt HIV-1 LTR-promoter mRNA (top sequence in red: 5′–3′). The cleavage site is indicated by the arrow. All the three modified nucleosides (A, C, and U) are in blue. (B and C) Multiple-turnover activities at 30 °C (left panel) and 37 °C (right panel), respectively. (B) Representative autoradiographic images of *trans*-cleavage activity ([D7-38-32t] = 5 nM, [substrate] = 500 nM); (C) Multiple-turnover profiles generated by fitting obtained *k*_obs_ values and substrate concentrations in Michaelis–Menten equation. Data are the mean of three replicative experiments, with *R*^2^ ≥ 0.98 in both cases.

By testing substrates of various length, we found optimal cleavage with a 19-nt substrate (an extra G was added to the 5′-terminus and an extra C was added to the 3′-terminus). Under simulated physiological conditions (0.5 mM Mg^2+^, 150 mM KCl, pH 7.5), Dz7-38-32t cleaved the 19 nt all-RNA substrate at the rGrU junction within the four-base bulge (5′-r(CCGU)-3′) that was positioned opposite the catalytic core. Cleavage proceeded with multiple turnover (Fig. 3A). Using 5 nM of Dz7-38-32t, values of *k*_obs_ were obtained for a range of substrate concentrations that were at least in 10-fold excess of Dz7-38-32t and which spanned *K*_M_. These data fit well to the Michaelis–Menten equation. At 30 °C and 37 °C, Dz7-38-32t attained *k*_cat_ values of 1.06 ± 0.08 min^–1^ and 0.27 ± 0.02 min^–1^, and *K*_M_ values of 1.37 ± 0.24 μM and 3.3 ± 0.55 μM, respectively, corresponding to second order rate constants (catalytic efficiencies) of 7.7 × 10^5^ M^–1^ min^–1^ and 8.2 × 10^4^ M^–1^ min^–1^, respectively (Fig. 3B and C). These *k*_cat_ values are 3–4 orders of magnitude greater than those of unmodified DNAzymes when evaluated at 0.5 mM Mg^2+^.

Based on the electrophoretic mobility of the 5′-^32^P-labeled cleavage product compared to bands generated with a carbonate ladder, it appears that the product contains a 2′-3′-phosphodiester at the 3′ terminus. Moreover, T4 polynucleotide kinase treatment of the reaction products with γ-^32^P-ATP gave a second (new) labeled fragment corresponding in length to the 3′-product, from which we inferred that the cleavage reaction involves transphosphorylative cleavage with 2′-anchimeric assistance rather than phosphodiester hydrolysis (data not shown).

Increasing the temperature from 30 °C to 37 °C gave a more than 2-fold increase in *K*_M_, a 4-fold drop in *k*_cat_, and an 8-fold reduction in catalytic efficiency overall. This is not unexpected as a temperature increase will increase the substrate dissociation rate thereby destabilizing the DNAzyme–substrate complex. In addition, greater thermal motion within the catalytic domain itself can reduce the apparent *k*_cat_ irrespective of the affinity for substrate.^44^ Increasing the length of the guide arms to accommodate two longer substrates (21 nt and 25 nt) in the hope of enhancing the stability of the DNAzyme–substrate complex did not improve activity at 37 °C. Notably, this effect has been seen in other DNAzymes and ribozymes and may be attributed to increased ground-state stabilization.

### Substrate specificity

To gain insight into the substrate specificity of Dz7-38-32t, three additional substrates were constructed. Each substrate contained a different ribonucleoside at the cleavage site, with the remainder of the sequence being unchanged. Multiple-turnover cleavage assays revealed that Dz7-38-32t displayed ∼20-fold reduced activity for a 5′-rArU-3′ junction, and <5% cleavage at either 5′-rUrU-3′ or 5′-rCrU-3′ junctions (Fig. S5, ESI†) with a small envelope of cleavage products. When a nearly all-DNA substrate was used, wherein the four unpaired nucleosides (CCGU) were riboses, *k*_cat_ and *K*_M_ values were each slightly depressed by a factor of 2–3. (See Fig. S6, ESI†).

When we undertook this selection, we appreciated that the 3′-guide arm would necessarily contain modifications, in this case 3 or 4 dC^aa^s along with one dA^im^. Previously, we showed that the dC^aa^s greatly stabilize complementary interactions, which we anticipated would constitute an advantage in terms of substrate recognition and would offset the slightly destabilizing effects of the dA^im^.^72^ Nevertheless, we also recognized that these modifications would limit the ability to alter the 3′-guide arm sequence. To test this, we replaced the dA^im^ in the guide arm with dG; the resulting DNAzyme had limited yet detectable activity against the complementary substrate (∼20%), and very little activity against the original substrate that would contain a G-U wobble mismatch (see Fig. S7, ESI†). This shows the potential for some mutability in terms of changing the guide-arms. Although we did not fully examine the extent to which the guide-arms could be altered to recognize other targets, we took this key finding as evidence that modified nucleosides in the guide arms provide contextual specificity for the target sequence used in the selection. Yet by the same token, the guide arms are less mutable for targeting other sequences (see Discussion section).

### Dependence of activity on divalent metal ions

Various divalent metal cations were investigated to determine their effect on the *trans*-cleavage rate of Dz7-38-32t under multiple-turnover conditions at 30 °C; Mg^2+^ and Ca^2+^ were tested due to their high physiological relevance, while Zn^2+^, Cu^2+^, and Hg^2+^ were examined because of their high affinity for the appended functionalities, in particular imidazole, even though the free intracellular concentrations of these divalent metal cations are negligible. Finally, Mn^2+^ and Pb^2+^ were investigated because of the high relevance of these ions to the activity of other reported DNAzymes. The obtained values of *k*_obs_ revealed Dz7-38-32t's tolerance to most of these metals: the presence of at least up to 10 mM Mg^2+^ or 0.5 mM of Ca^2+^, Zn^2+^, Cu^2+^, and Mn^2+^ caused only negligible differences in initial velocity values suggesting a robust, M^2+^-tolerant activity yet M^2+^-independent (or perhaps M^2+^-indifferent) folding adopted by Dz7-38-32t. The presence of 100 μM Pb^2+^ or 50 μM Hg^2+^ caused a significant decrease in activity, which is not unexpected due to the well-known affinity that amines and imidazoles have for these cations. That these divalent metal cations, particularly Hg^2+^, inhibited this DNAzyme further corroborates the importance of amines, and more likely imidazoles for structure or function or both (Table 1, and Fig. S8, ESI†).

**Table 1:** Effects of divalent metal ions on *trans*-cleavage reaction of Dz7-38-32t^*a*^

M^2+^	Concentration (mM)	*k*_obs_ (min^–1^)
M^2+^-free	0	0.43
Mg^2+^	0.5	0.51
Cu^2+^	0.5	0.36
Zn^2+^	0.5	0.41
Ca^2+^	0.5	0.46
Mn^2+^	0.5	0.48
Pb^2+^	0.05	0.26
Hg^2+^	0.05	0.10

^*a*^All the reactions were carried out in 50 mM cacodylate buffer (pH 7.45) containing 150 mM KCl, and the tested concentration of M^2+^, at 30 °C. [Dz] = 5 nM, [S] = 1 μM.

### Dependence of activity on pH

The pH dependence of Dz7-38-32t was studied under multiple-turnover conditions over the pH range of 6.0–8.5 at saturating conditions. The log(*k*_obs_) value increased linearly in the pH range of 6.0–7.0 with a slope of 1.14 and decreased in the pH range of 7.0–8.5 with a negative slope value of –1.1 (Fig. S9, ESI†) giving a bell-shaped pH-rate profile. This profile suggests that base catalyzed 2′OH deprotonation at low pH values or acid catalysed 5′OH protonation at higher pH is rate-limiting. Such a profile is consistent with a concerted SN2-like transesterification reaction typified by RNase A. Moreover, fitting *k*_obs_ values to eqn (S3) (see ESI†) suggests two ionizable groups with two p*K*_a_s (p*K*_a1_ and p*K*_a2_), each calculated to be nearly 7.0. While the appearance of a bell-shaped pH-rate profile is consistent with the hypothetical mechanism involving two ionizable groups, most likely two pendant imidazoles, it is not impossible that one of the nucleobases could provide similar catalytic functionality due to a perturbed p*K*_a_. Alternatively, conformational changes along with the possibility of other p*K*_a_ perturbed groups may account for the pH-rate profile we observed. Such kinetic ambiguity has been observed for naturally occurring ribozymes. Since many ionisable groups can also influence folding, we should apply caution in concluding that the bell-shaped rate profile necessarily reflects an active site analogous that of RNase A.

## Discussion

The ability to target RNA for catalytic destruction is a goal that has been approached by many investigators. Particularly impressive has been the use of combinatorial selection techniques for new DNAzymes with the potential for folding into a catalytic motif that is situated between two guide-arms that enable sequence-specific binding that precedes catalysis. Whereas many DNAzymes have been selected to cleave at a single embedded ribophosphodiester linkage, most of these DNAzymes are inept at cleaving a homologous all-RNA substrate. Modified DNAzymes, which we previously reported, proved to be no exception.

To wit, Dz10-66,^70^ a 45-nt catalyst that cleaved a substrate with a single ribophosphodiester linkage with multiple turnover, and Dz12-91,^69^ a 19-nt self-cleaving species that cleaved a 17-nt substrate containing twelve ribophosphodiester linkages could not cleave an all-RNA substrate of the same sequence used herein. These catalytic shortcomings provided an impetus for the *de novo* re-selection herein that was required to test whether modified dNTPs could be applied towards the selection of catalysts that cleave all-RNA targets, an endeavor for which success could not be predicted *a priori* or easily adapted from these antecedent catalysts. Not surprisingly, Dz7-38-32 showed no apparent homology to either aforementioned catalyst.

Nucleoside triphosphates dA^im^TP, dC^aa^TP, and dU^ga^TP (**1–3**) had been designed to satisfy two critical objectives: (1) to introduce key chemical functionalities into DNA that are typically found at the active sites of nucleases; (2) to preserve the Watson–Crick faces that are needed for sequence-specific insertion and faithful recopying by DNA polymerases. Recently, we demonstrated that Sequenase, the enzyme used to prepare Dz7-38-32, inserts **1–3** with high fidelity while Taq recopies the resulting modified strands with equally high fidelity resulting in less than 1% mutation overall.^72^ That insertion and read-out result in the selection of a given DNAzyme containing **1–3**, reflects the incontrovertible consequences of semiconservative replication along with Chargaff's rules, which ensure that a modified nucleoside will either be perpetuated through rounds of selection or otherwise irretrievably lost. More importantly, we demonstrated that the enzymatic synthesis of these DNAzymes provides homogeneous species that are as pure as any other enzymatically synthesized DNAzyme or ribozyme.

Herein, we have selected two DNAzyme families that self-cleave at a 17 nt all-RNA target in the absence of a divalent metal cation (M^2+^). Notably, we identified these families starting with two cloned sequences obtained in the 7th round of a naive selection. By round 7, the apparent self-cleavage activity had reached a maximum level and there was no increase in activity in round 8 and 9. After subjecting two clones to chemical re-synthesis at 15% degeneracy, we conducted several more rounds of re-selection that resulted in a significant increase in activity, and ultimately giving these two families from which two sequences were chosen for characterization.

Dz7-38-32, exhibited an exceptionally high rate constant for self-cleavage (4.9 min^–1^). To the best of our knowledge, this is the highest rate constant ever observed for M^2+^-free self-cleavage, and is up to 5 orders of magnitude greater than that seen for unmodified catalysts capable of the same reaction under similar conditions. Indeed, this value rivals those found in some of the most proficient M^2+^-dependent self-cleaving DNAzymes. While this rate constant is identified in the context of biphasic kinetics, a second rate constant of 0.06 min^–1^ is still appreciable for M^2+^-independent cleavage.

The apparent biphasic kinetics suggest the presence of at least two conformations, which self-cleave according to defined rate constants for each phase. Other explanations include the existence of a kinetically inactive fold that refolds slowly to the active phase, as well as the possibility of an internal equilibrium with the active site whereby re-ligation contributes to a reverse reaction.^73^ Indeed, such biphasic kinetics have been observed for DNAzymes^74^–^76^ and ribozymes,^77^ including the hairpin ribozyme, which was engineered for intermolecular cleavage and advanced for therapeutic applications despite the existence of such kinetics.

In light of these high rate constants, we re-engineered Dz7-38-32 for intermolecular cleavage; Dz7-38-32t showed all of the characteristics of an enzyme, including high substrate (sequence) specificity, saturation kinetics, and multiple turnover with high apparent *k*_cat_ values (>1 min^–1^ at 30 °C and 0.27 min^–1^ at 37 °C). The pH-rate profile for Dz7-38-32t shows a standard bell-shape suggesting that two functionalities with p*K*_a_s near neutrality play the roles of general base and acid catalysis. With an abundance of imidazoles, it is likely that two of these play critical roles in catalysis, although other functionalities with perturbed p*K*_a_s, including the nucleobases themselves, may also be capable of the same. Interestingly, the presence of Mg^2+^, or other divalent metals, except Hg^2+^, did not diminish catalytic activity, suggesting that the catalytically competent fold or folds are not perturbed by the re-introduction of these cations.

In terms of sequence specificity, Dz7-38-32t showed >20-fold selectivity for the target sequence compared to targets which differed in terms of the nucleobase located 5′ with respect to the cleavage site. These results stand in contrast to Dz10-23, which showed higher *k*_cat_ values for a substrate where the unpaired nucleoside at the 5′-cleavage was changed from the native A to a G.

To assess the potential for reengineering Dz7-38-32t to cleave new targets, we varied the 3′-guide arm and observed limited success; replacement of dA^im^ with dG resulted in the expected preferential recognition of a target C over the original U, yet not without significant catalytic impairment. This is curious since *a priori* the dG would be expected to be more stabilizing than the dA^im^, not only due to an additional H-bond but also because dA^im^ was shown to be destabilizing to DNA duplexes compared to dA.^72^ While the exact basis for the catalytic impairment with a dG *in lieu* of dA^im^ remains unclear, we surmise that the dA^im^ may destabilize the ground-state with respect to the transition state while dG effects the reverse. Alternative explanations exist; for example, modified nucleosides will introduce subtle, long-range helical distortions in the ground state that propagate through the transition state to enable the catalyst to force the 2′OH of the target ribose in line with the scissile phosphate. Such distortions are altered when the modified nucleosides are replaced with different ones.

The presence of modifications in the 3′-guide arm are necessarily a consequence of the selection methodology. While these are expected to impart the advantage of sequence specificity, at least in the context of the selection, these modified nucleosides may limit the potential for re-engineering a duly selected DNAzyme to recognize different targets. Notably, such limitations still befall unmodified DNAzymes; to wit, when Dz10-23 was re-engineered to cleave the *env* mRNA, it showed a 3-fold reduction in *k*_cat_ and a 3000-fold reduction in *k*_cat_/*K*_M_.^43^ Our data recapitulate this well-established phenomenon, namely that alterations in guide-arm composition may give rise to large differences in both *k*_cat_ and *K*_M_ values. In our case, these differences may be magnified due to the contextual effects of the added modifications in the guide arms.

The flexibility to alter target specificity by simply changing the guide arms represents an advantage in DNAzyme engineering that may be compromised by the use of modified bases. Yet such flexibility is not a precondition for therapeutic applications. Instead, specificity for a given target must be the primary focus, as has always been the case in aptamer development, including high affinity modified ones,^78^–^81^ for which each target requires a different selection. Accepting this outlook, we contend that combinatorial selections using **1–3** offer a vast reservoir for discovering RNase A mimics, some of which should be highly specific for a given pathogenic mRNA target against which they would be selected. It has not escaped our attention that **1–3** may be also used to select aptamers, which naturally bind ground states rather than transition states. We expect such aptamers will possess an enhanced ability to recognize anionic targets and function competently in the absence of Mg^2+^. Such would in turn enable recognition of targets that cannot be recognized in the presence of Mg^2+^*e.g.* nucleases.

## Experimental

DNA oligonucleotides and chemically modified triphosphates. dA^im^TP and dU^ga^TP were synthesized according to literature precedent,^71^,^82^ and dC^aa^TP was obtained from Trilink. Oligonucleotides were purchased from Integrated DNA Technologies (IDT), and purified by denaturing (7 M urea) 10 or 20% polyacrylamide gel electrophoresis (PAGE) before use. Details regarding the oligonucleotide sequences and the preparations are provided in the ESI.† α-^32^P-dGTP and γ-^32^P-ATP were purchased from Perkin Elmer.

### *In vitro* selection

Approximately 15 pmol of modified DNA was used into each round of selection. DNAs were internally labeled by adding trace amount of α-^32^P-dGTP (10–15 μCi) during template-directed primer extension reaction. Briefly, 15 pmol of synthetic DNA template was hybridized to 15 pmol of 5′-biotinylated primer containing the all-RNA target sequence. Sequenase was used to incorporate dA^im^TP, dC^aa^TP and dU^ga^TP along with dGTP (50 μM dA^im^TP, 25 μM dC^aa^TP, 10 μM dU^ga^TP and dGTP) at 32 °C for 4 h. The reaction was then quenched by the addition of 2 μL of EDTA (0.5 M, pH 8.0) to a final concentration of ∼25 mM. Following standard removal of the template strand by 5 brief washes with 0.1 M NaOH, the initial pool of modified DNA was allowed to self-cleave in cleavage buffer containing 50 mM sodium cacodylate (pH 7.5), 200 mM NaCl, and 1 mM EDTA for 1 h. Cleaved DNAs were separated and purified by denaturing 7% PAGE. The purified modified DNA was PCR amplified by Vent (*exo*-) DNA polymerase to generate double-stranded DNAs. The non-template strands generated by using a 5′-phosphorylated primer was digested using λ exonuclease, and the template strands were purified by denaturing 10% PAGE and used into the following round of selection. The selection stringency was gradually increased by shortening cleavage reaction time, or decreasing the concentration of NaCl in cleavage buffer, or both.

To create the initial libraries of the two lineages of re-selection, random mutations at a frequency of 15% to each of the nucleoside positions encompassing the catalytic core were introduced to two best-acting clones out of the selection process, namely Dz7-45 and Dz7-38, respectively. Mutagenic PCR was performed to increase sequence diversity during the process of re-selection. A detailed description of the selection, re-selection and their respective enrichment progressions are included in the ESI.†

### Intramolecular DNAzyme cleavage assays (*in cis*)

*cis*-Cleaving DNAzymes were prepared in the same way as the modified libraries for selection. Approximately 15 pmol of 5′-biotinylated primer containing the all-RNA target was annealed to the synthetic template of a corresponding DNAzyme. Then Sequenase was used to medicate the polymerization of dA^im^TP (50 μM), dC^aa^TP (25 μM) and dU^ga^TP (10 μM) along with dGTP (25 μM) at 32 °C for 4 h. Trace amount of α-^32^P-dGTP was used for internal labeling of DNAzymes. The EDTA (25 mM) quenched extension product was immobilized on 100 μL of pre-washed streptavidin coated magnetic beads by incubating at room temperature for 15 min. The template strand was then removed by five quick washes of 100 μL NaOH (0.1 M and 1 mM EDTA). The resulting modified strand sticking to streptavidin beads was immediately neutralized by 200 μL of neutralization buffer (25 mM cacodylate, pH 6) followed by a final 100 μL of water wash. Then the modified DNA immobilized on beads was subjected to self-cleavage reaction in 100 μL of standard cleavage buffer (50 mM cacodylate pH 7.45, 200 mM NaCl, 1 mM EDTA). 5 μL of the mixture was removed and quenched in 15 μL of formamide (containing 1 mM biotin, 25 mM EDTA, 0.01% bromophenol blue and 0.01% xylene cyanole) at certain time points over a period of 150 min. Samples were resolved by denaturing 7% PAGE, visualized and quantified by PhoshporImager. Values of *k*_obs fast_ and *k*_obs slow_ were calculated using a double-exponential equation:
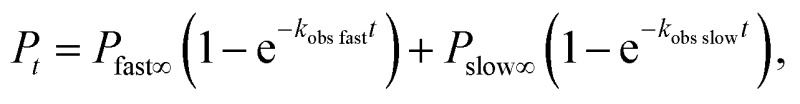
where *P*_fast∞_ and *P*_slow∞_ are the respective amplitudes of the two cleavage phases, *P*_*t*_ is the total fraction cleaved at time *t*, and *k*_obs fast_ and *k*_obs slow_ are the observed rate constants of the two cleavage phases, respectively.

### Intermolecular DNAzyme cleavage (*in trans*)

*trans*-Acting Dz7-38-32t was enzymatically synthesized by application of the same conditions used for the synthesis of *cis*-acting DNAzymes in *in vitro* selection. Differently, a synthetic template containing a 5′-biotin moiety along with a truncated DNA primer corresponding to the 3′-end of the hairpin loop in Fig. 1B were used for template-directed primer extensions. Dz7-38-32t was separated from the template which remained immobilized on streptavidin beads using 0.1 M NaOH, followed by an immediate neutralization with 0.1 M HCl to a pH ∼ 7.5. The resulting DNAzyme was further desalted using G-25 spin column which removed the salt and provided pH equilibration in 10 mM Tris–HCl. The quantity of DNAzyme produced was determined by relating autoradiographic density to signal volume and ultimately to the number of pmol of α-^32^P-dGTP as previously reported.^70^

To determine values for *k*_cat_ and *K*_M_, multiple turnover cleavage assays were performed in the presence of 5–10 nM Dz7-38-32t and a range of all-RNA substrate concentrations that were in at least 10-fold excess of Dz7-38-32t exceeding *K*_M_. The reaction rate (*v*_obs_) for each substrate concentration [S] was calculated by linear fitting at least five data points obtained over the first 10–20% of cleavage reaction. *k*_cat_ and *K*_M_ values were determined by plotting the *v*_obs_ values *versus* substrate concentrations to the Michaelis–Menten equation:
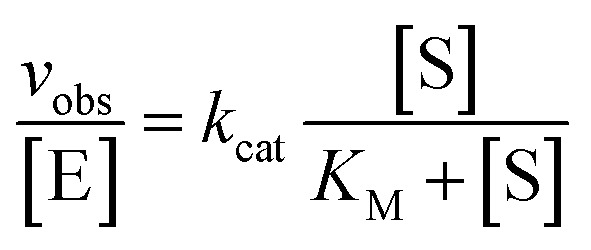
where [E] represents the DNAzyme concentration, which was 5 nM in the assays here. For pH and metal ion dependence assays, these parameters were altered accordingly.

## Conclusions

In summary, we have successfully selected a new generation of M^2+^-independent RNA-cleaving DNAzymes through *in vitro* selection using dA^im^TP, dC^aa^TP, and dU^ga^TP. In contrast to previous reports from our lab on DNAzymes that were incapable of cleaving an all-RNA target, herein we now report fully active DNAzymes that cleave an all-RNA substrate with high efficiency and sequence-specificity. A systematic characterization of *trans*-acting Dz7-38-32t revealed *k*_cat_ and *K*_M_ values of 0.27 min^–1^ and 3.3 μM under simulated physiological conditions (pH 7.45, 150 mM K^+^, 0.5 mM Mg^2+^, 37 °C), corresponding to a catalytic efficiency of ∼10^5^ M^–1^ min^–1^. Although this value is still far from catalytic perfection, this represents the highest second order rate constant achieved by any nucleic acid catalyst (RNA or DNA) when evaluated under low-M^2+^ characteristic of cellular conditions up to date.

It is notable that the three added functionalities have all been used in other contexts to improve the properties of antisense oligonucleotides in terms of increased nuclease stability and enhanced cell penetration. Such properties may further enhance the applications of similarly modified DNAzymes. Bio-stability, local divalent cation concentration, efficient cellular uptake, as well as the subcellular localization are all factors that will influence the eventual therapeutic applications of DNAzymes. In this regard, the demonstrated M^2+^-independence, efficient multiple catalytic turnover, along with the potential for cell penetration may further overcome several limitations that restrict the *in vivo* application of unmodified DNAzymes. A scalable chemical synthesis of Dz7-38-32t to fully address its intracellular activity and toxicity profile are worthy of systematic investigation. Such goals are currently under way. In the meantime, we expect that this report brings us closer to realizing the therapeutic promise of catalytic nucleic acids that had been articulated in the seminal literature decades ago.

## Conflicts of interest

There are no conflicts to declare.

## Supplementary Material

Supplementary information
