# Supplementary material for: A densely modified M2+-independent DNAzyme that cleaves RNA efficiently with multiple catalytic turnover
Source: Chem Sci. 2018 Jan 16;9(7):1813–21. doi: 10.1039/c7sc04491g (PMC5890787; doi:10.1039/c7sc04491g)
Supplement: Supplementary file 1 [file SC-009-C7SC04491G-s001.pdf]

Electronic Supplementary Material (ESI) for Chemical Science.
This journal is © The Royal Society of Chemistry 2018

Supplemental Information

A densely modified M^{2+}-independent DNAzyme that cleaves RNA efficiently with multiple catalytic turnover

Yajun Wang, Erkai Liu, Curtis H. Lam, David M. Perrin*

Department of Chemistry, University of British Columbia, 2036 Main Mall, Vancouver, British Columbia, Canada, V6T 1Z1

*To whom correspondence should be addressed. Email: dperrin@chem.ubc.ca

Table of Contents

General materials S3
Oligonucleotides S3-S5
Buffers S5
In vitro selection S6-S9
    Template-directed primer extension to transcribe modified library for selection S6
    Selection S6, S7
    Cloning of cDNA S7
    Single-clone activity screening S7, S8
    Re-selection S8-S10

Cleavage activity *in cis* S11-S13
    Kinetic activity *in cis* S11, S12
    Testing the need for modifications S13

Cleavage activity *in trans* S14-S22
    Enzymatic synthesis of Dz7-38-32t S14-S16
    General protocol for trans-cleavage reactions S17
    Characterization of k_{cat} and K_M with the optimal 19 nt all-RNA S17
    Base preference at cleavage site and substrate generality S18
    Activity of Dz7-38-32t on the chimeric DNA/RNA substrate S19
    The function of dA^{im} in the modified 3' substrate-recognition arm S20
    Effects of divalent metal ions on *trans*-cleavage reaction of Dz7-38-32t S21
    The pH dependence study of Dz7-38-32t S22

References S23

General materials.

$dA^{im}TP$[1] and $dU^{ga}TP$[2] were synthesized according to literatures, respectively. $dC^{aa}TP$ was obtained from TriLink Bio Technologies. All oligonucleotides were purchased from Integrated DNA Technologies (Coralville, IA), and purified by 10-20% denaturing PAGE (7 M urea). Ultrapure dNTPs were obtained from Fermentas. Vent exo^- DNA polymerase, *Taq* DNA polymerase, lambda exonuclease, and T4 polynucleotide kinase were obtained from New England Biolabs. Sequenase V2.0 and pyrophosphatase were purchased from Affymetrix. γ-^{32}P-ATP and α-^{32}P-dGTP were purchased from Perkin Elmer. Sephadex G25 resin was obtained from GE. Streptavidin magnetic particles were purchased from Roche.

Oligonucleotides (ON, shown 5' to 3').

1 Selection library containing 40 randomized positions, **ON 1**:
GCGCTCGCGCGGCGTGCN_{40}CTGTTGGCGCAGGCCGACGC.

2 Primer for in vitro selection and *cis*-cleavage characterization, **ON 2**:
Biotin-T_{40}CCCGGGTTTTT^r(GCGUGCCCGUCUGUUGG)TTTTGCGTCGGCC TGCGCCAACAG.

3 PCR amplification primers:
GCGCTCGCGCGGCGTGC, P2 (**ON 3**).
phosphate-GCGTCGGCCTGCGCCAACAG, P3 (**ON 4**).
TTTTTTTTTTTTTTTTTTTTGCGCTCGCGCGGCGTGC, P4 (**ON 5**).
phosphate-TTTTGC GTCGGCCTGCGCCAACAG, P5 (**ON 6**).

4 Template for transcription of *cis*-acting Dz7-38-32, **ON 7**:
GCGCTCGCGCGGCGTGTACCATCCTGACGCTCCACACGTCCCAATCGATACC ACTGTAACTGTTGGCGCAGGCCGACGC.

5 Template for transcription of *cis*-acting Dz7-45-28, **ON 8:**
GCGCTCGCGCGGCGAAGGCCTGACTCAGGCACTCCGCATCCATCCGTCCGC TGCTTCTGTTGGCGCAGGCCGACGC.

6 Template for transcription of *cis*-acting Dz7-38-39, **ON 9:**

GCGCTCGCGCGGCGTGTACCATCCTGACGCTCCACACGTCCCAATCGATACC
ACTGTAACTGTTGGCGCAGGCCGACGC.

7 Template for transcription of *cis*-acting Dz7-38-83, **ON 10:**
GCGCTCGCGCGGCGTGATCCCATCCTGACGCTACACACGTCCCAATCGATAC
CACTGTAACTGTTGGCGCAGGCCGACGC.

8 Template for transcription of *cis*-acting Dz7-38-90, **ON 11:**
GCGCTCGCGCGGCGTGATCCCATCCTGACGCTCCACACGTCCCAATCGATAC
CACTGTAACTGTTGGCGCAGGCCGACGC.

9 Template for transcription of *cis*-acting Dz7-45-10, **ON 12:**
GCGCTCGCGCGGCGAAGCCCTACTCGGGACTCCGCGTCACACCACATCCGC
TGCTTCTGTTGGCGCAGGCCGACGC.

10 Template for transcription of *cis*-acting Dz7-45-22, **ON 13:**
GCGCTCGCGCGGCGAAGCCCTACCCGGGACTCCGCGTCACACCACATCCGC
TGCTTCTGTTGGCGCAGGCCGACGC.

11 Primer for transcription of Dz7-38-32t, **ON 14:**
GCCTGCGCCAACAG.

12 Template for transcription of *trans*-acting Dz7-38-32t, **ON 15**:
Biotin-GCGCGGCGTGCTGTACCATCCTGACGCTCCACACGTCCCAATCGAT
ACCACTGTAACTGTTGGCGCAGGC.

13 Template for transcription of *trans*-acting Dz7-45-28t, **ON 16:**
Biotin-GCGCGGCGAAGGCCTGACTCAGGCACTCCGCATCCATCCGTCCGCT
GCTTCTGTTGGCGCAGGC.

14 Original 19 nt all-RNA substrate, **ON 17**:
^{r}(GGCGUGCCCGUCUGUUGGC).

15 19 nt all-RNA substrate containing mutated RNA base at the cleavage site:
^{r}(GGCGUGCCC$\underline{A}$UCUGUUGGC) **ON 18**, ^{r}(GGCGUGCCC$\underline{C}$UCUGUUGGC) **ON 19**, and ^{r}(GGCGUGCCC$\underline{U}$UCUGUUGGC) **ON 20**.

16 19 nt chimeric DNA/RNA substrate, **ON 21**:
GGCGTGC^{r}(CC$\underline{G}$U)CTGTTGGC.

17 The guide arm U-to-C mutated substrate, **ON 22:**
^{r}(GGCG$\underline{C}$GCCCGUCUGUUGGC).

"N_{40}" in **ON 1** represents 40 randomized positions. "r" designates a stretch of RNA bases. Underlined bases in all different versions of substrates represent the mutated base relative to the original one.

Buffers.

1 1X T4 polynucleotide kinase buffer: 70 mM Tris-HCl (pH 7.6 @ 25°C), 10 mM $MgCl_2$, 5 mM DTT.

2 1X Sequenase buffer: 40 mM Tris-HCl (pH 8.0), 50 mM NaCl, 20 mM $MgCl_2$.

3 Gel loading buffer: formamide, 25 mM EDTA, 0.01% bromophenol blue and 0.01% xylene cyanole.

4 Gel loading buffer containing biotin: gel loading buffer/100 mM biotin in DMF (99:1).

5 Wash buffer (TEN): 50 mM Tris-HCl (pH 7.5), 200 mM NaCl, 1 mM EDTA.

6 Neutralization buffer: 25 mM sodium cacodylate (pH 6.0), 1 mM EDTA.

7 1X *cis*-cleavage buffer: 50 mM sodium cacodylate (pH 7.4), 200 mM NaCl, 1 mM EDTA.

8 10X *trans*-cleavage buffer (containing Mg^{2+}): 0.5 M sodium cacodylate (pH 7.4), 1.5 M KCl, 5 mM $MgCl_2$.

9 10X M^{2+}-free *trans*-cleavage buffer: 0.5 M sodium cacodylate (pH 7.4), 1.5 M KCl.

10 5X pH variant *trans*-cleavage buffer: pH 6.0-7.0, 250 mM sodium cacodylate, 750 mM KCl, 2.5 mM $MgCl_2$; pH 7.5-8.5, 250 mM sodium phosphate, 750 mM KCl, 2.5 mM $MgCl_2$.

11 M^{2+} stock solutions in H_2O: 5 mM $CuCl_2$, $ZnCl_2$, $CaCl_2$, and $MnCl_2$; 0.5 mM $Pb(CH_3CO_2)_2$ and $Hg(CH_3CO_2)_2$.

12 Gel elution buffer: 10 mM Tris-HCl, (pH 8.0), 1% $LiClO_4$.

13 1X TBE solution: 89 mM Tris base, 89 mM boric acid, 2 mM EDTA.

14 5X modified nucleotides cocktail: 250 μM $dA^{im}TP$, 125 μM $dC^{aa}TP$, 50 μM $dU^{ga}TP$, 50 μM dGTP.

***In vitro* selection.**

Template-directed primer extension to transcribe modified library for selection. 15 pmole of template library (**ON 1**) and selection primer (**ON 2**), 8 μL of 5X Sequenase buffer, and H_2O were combined to a volume of 26.5 μL, and heated to above 95°C for 5 min. The solution was then left at room temperature to slowly cool down to room temperature to anneal template and primer. Following that, reagents including 8 μL of 5X modified dNTP cocktail (250 μM $dA^{im}TP$ 125 μM $dC^{aa}TP$, 50 μM $dU^{ga}TP$, and 50 μM dGTP), 2 μL of DTT (100 mM), 1 μL of pyrophosphatase, 1.5 μL of α-^{32}P-dGTP (~15 μCi), and 1 μL of Sequenase v2.0 were added sequentially to the annealing solution. The reaction was then mixed well and covered with 20 μL of mineral oil. The polymerization reaction was run at 32°C for 4 h and then quenched by the addition of 2 μL of EDTA (0.5 M, pH 8.0) to a final concentration of ~ 25 mM.

Selection. 50 μL of streptavidin beads was magnetized, decanted, and subjected to three washes with TEN buffer (100 μL per wash). The primer extension product was incubated with the washed beads for 15 min at room temperature to immobilize the DNA duplex on beads. Following two more washes with 100 μL of TEN after immobilization, the template strand was stripped away by five quick washes (no longer than 30 seconds per wash) with 100 μL of 0.1 M NaOH containing 1 mM EDTA. The resulting modified strand sticking on streptavidin beads was immediately neutralized using 200 μL of neutralization buffer, followed by a final 100 μL of DEPC-treated sterile water wash. The modified DNA library on beads was then allowed to fold and self-cleave in 100 μL of cleavage buffers of different ionic strength for varying time spans with the progress of *in vitro* selection. After controlled reaction time, the 100 μL of cleavage reaction was magnetized, of which only 90 μL of the supernatant was recovered to minimize the contamination caused by the agitation of magnetic beads, precipitated by 1% $LiClO_4$ in acetone, re-suspended in gel loading buffer, and purified by 7% denaturing PAGE. The purified self-cleavage product was amplified twice: the first amplification (using primer set **ON 3**/**ON 4**) was

used to “reverse-transcribe” the modified DNA strand into unmodified one, and the second amplification (**ON 5**/**ON 6**) was in prep-scale to provide template for the following round of selection. Lambda exonuclease was used to digest the 5’-phosphorylated non-template strands, which were amplified on the phosphorylated primers (**ON 4** and **ON 6**), respectively. Trace amount of α-^{32}P-dGTP was used to internally label the amplicons of the first amplification step, and the amplicons of the second amplification step were visualized by UV-shadowing, which were both subjected to PAGE purification after Lambda exonuclease digestion.

Cloning of cDNA. The population collected after 0.5 min of cleavage reaction from G7 (indicated by the arrow) of selection was amplified using *Taq* DNA polymerase with thermal cycles containing a final extension step of 30 min to generate PCR products with 3’-A overhangs. The amplicons were purified on a 2% agarose gel using GeneJet Gel Extraction Kit before they were TOPO cloned into the pCR2.1-TOPO vector according to the manufacture’s instruction. The vector was then used to transform *E.coli* DH5α following a normal chemical transformation protocol. White transformed colonies were picked by Blue-White screening on LB Agar plate containing 100 mg/L ampicillin for inoculation. Plasmids were prepared by using PureLink Quick Plasmid Miniprep Kit (Invitrogen) followed by fragment insertion check by endonuclease *EcoR I* digestion. Plasmids containing a single insertion of the amplicon were sequenced using M13R primer by Nucleic Acid Protein Service Unit of UBC (NAPS-UBC).

Single-clone activity screening. Synthetic oligonucleotides corresponding to the various clones were used as templates to synthesize modified DNA as described previously in “**Template-directed primer extension to transcribe modified library for selection”**. Consequently, 5 pmol of each individual synthetic oligonucleotide were then immobilized on streptavidin magnetic particles. Following the same procedures described in “**In vitro selection**”, the single-stranded modified DNAs were then incubated at room temperature in 60 µL of 1X cis-cleavage buffer **7**. At least 5 time points (10 µL each) were taken over a course of 180 min cleavage

reaction, and resolved by 7% denaturing PAGE (7 M urea). 11 clones showed self-cleavage activity among 58 clones that were screened, and were categorized into two families (**Table S1**).

Table S1 Active sequences isolated from the process of selection

Family	Clone (Dz)	Sequence (5' to 3', catalytic)
	7-10	TTACAGTGGTAGCGGTTGGCACGTGTGCAGCGTAAGTGGGCG
	**7-38**	TTACAGTGGTAGCGGTTGGCATGTGTGCAGCGTAGGTGGGCG
	7-44	TTACAGTGGTAGCGGTGGCATGTGTACAGCGTTAGTGAGTG
I	7-50	TTATAGTGGTAGCGGTTGGCATGTGTGTGGCGTGAGTTAGTG
	7-55	TTACAGTGGTAGCGGTTGGCATGTGTGGAGCGTAAGTGAGCG
	7-57	TTACAGTGGTAGCGGTTGGCATGTGTGCAGCGTAAGTGGGCG
	7-60	TTACAGTCGTAGCGGTGGCATGCGTGCAGAGTAAGTGGGCG
II	**7-45**	AAGCAGCGCATGTGATGGCACGCGTAGTGGCTGGGTAGCT

Only the sequence in the randomized region is shown from the 5' end to the 3' end. Letters in blue denote modified bases.

Re-selection. Two re-selections were initiated by partially randomizing the catalytic regions of Dz7-38 and Dz7-45. In detail, random mutations at a frequency of 15% per nucleoside position over all the positions that encompassed the catalytic center were introduced to the parental sequences. Thereby, two separate libraries containing ~0.1% and ~0.15% of their own parental sequences were built, respectively. Members of the two libraries were then challenged to perform all-RNA cleavage reaction by being subjected to 14 rounds of re-selection and amplification as described in above in "**Selection**". In addition to templates generated from amplifying the self-cleaved species using regular PCR protocol, templates from rounds 5, 8, 10 and 13 were supplemented with 10% of the DNA generated by amplifying the self-cleaved populations resulting from previous rounds following a mutagenic PCR protocol.[3] For both of the two re-selections, the populations collected after 5 min of cleavage reaction from G14 were *Taq* amplified, cloned, and screened for self-cleavage activity.

A

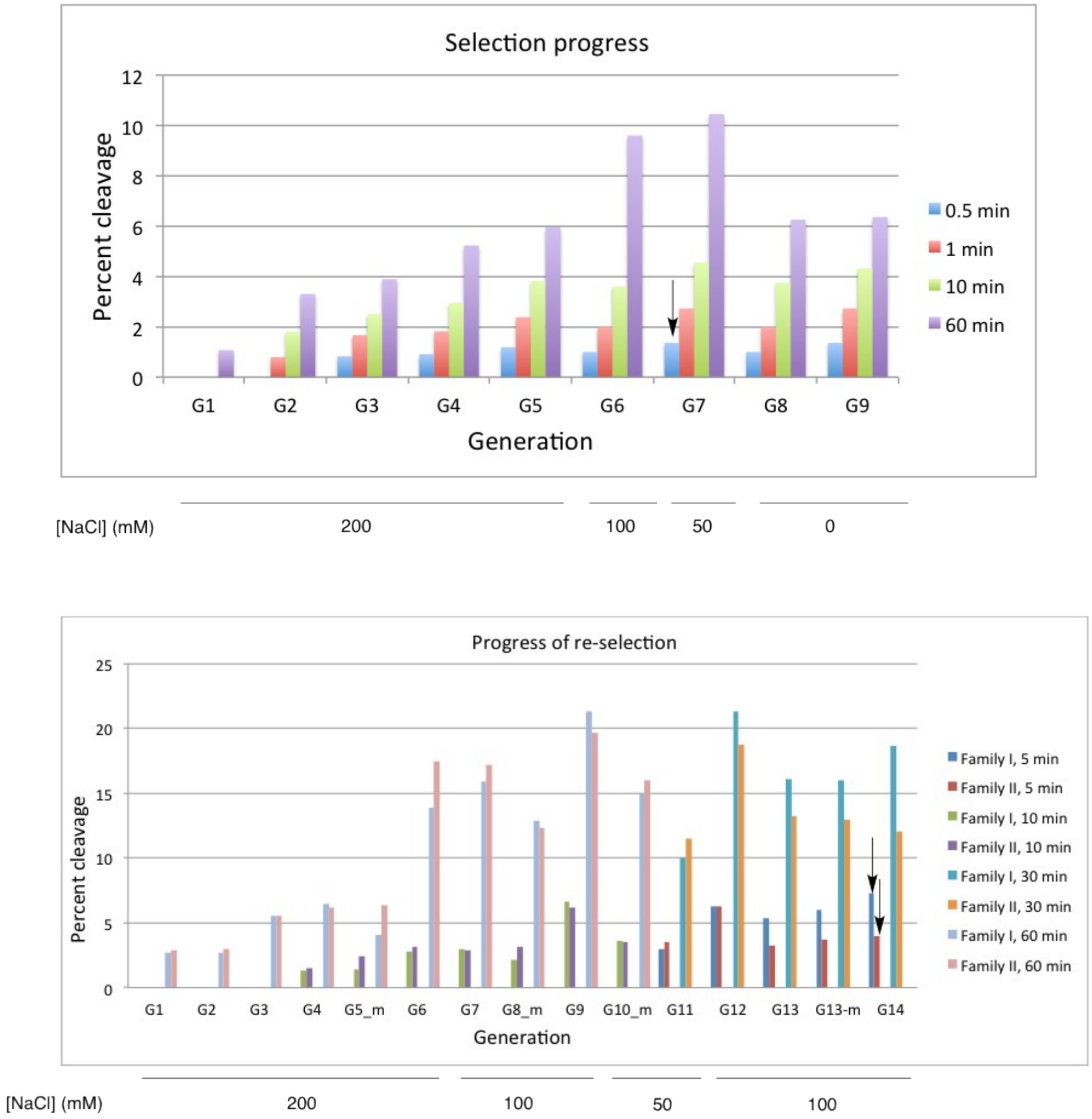

Figure S1. *In vitro* selection (**A**) and re-selection (**B**) progress. Plot of percentage of populations cleaved versus selection round (Gn). NaCl concentrations contained in cleavage buffers are labeled underneath the graph. Fast-to-cleave populations (indicated by arrows) collected after 0.5 min cleavage reaction of selection G7, and 5 min cleavage reaction of re-selection G14 were amplified and cloned.

Cleavage activity *in cis*.

Kinetic analysis *in cis*. Self-cleaving DNAzymes shown in Figure 2A were prepared in the same way as the modified library in the selection using respective synthetic

templates (**ON 7** to **ON 13**) ordered from IDT.

The extension product was immobilized on 100 µL of pre-washed (by TEN buffer, 100 µL per wash) streptavidin coated magnetic beads by incubating at room temperature for 15 min. Single-stranded DNAzyme was obtained in the same way as described previously. Then the DNAzyme on beads was incubated in 100 µL of standard cleavage buffer (50 mM cacodylate pH 7.45, 200 mM NaCl, 1 mM EDTA), which formed a mixture of uncleaved (sticking to streptavidin beads) and cleaved (falling off streptavidin beads into solution) materials. For Dz7-38-32, 5 µL of the mixture was removed and quenched in 15 µL of gel loading buffer containing 1 mM biotin at time points 0, 0.17, 0.33, 0.50, 0.67, 1, 1.5, 2, 3, 5, 10, 15, 20, 30, 45, 60, 75, 90, 105, 120, and 150 min, respectively. For Dz7-45-28, 5 µL of the mixture was removed and quenched in 15 µL of gel loading buffer containing 1 mM biotin at time points 0.50, 1, 3, 5, 10, 15, 30, 74, 118, and 150 min, respectively. All samples were denatured by incubating at 95°C for 5 min, followed by snap-cooling on ice before resolving the supernatant after magnetization on denaturing PAGE (7%). Visualizations and quantifications were carried out using Typhoon 9200 PhosphorImager (Amersham). The autoradiographic data of the cleavage reactions (represented by pixel volumes on the Imagequant software) were obtained by drawing polygons around the bands corresponding to the cleaved and uncleaved species. These data were fitted to a double exponential first-order reaction using Prism 6.

Equation S1: $P_t = P_{fast\,\infty}\left(1 - e^{-k_{obs\,fast}t}\right) + P_{slow\,\infty}\left(1 - e^{-k_{obs\,slow}t}\right)$,

where $k_{\text{obs fast}}$ and $k_{\text{obs slow}}$ are the observed *cis*-cleavage rates of the fast and slow phases, and the $\mathrm{P}_{fast\,\infty}$ and $\mathrm{P}_{slow\,\infty}$ are the amplitudes of the two phases, respectively.

A

Family I
Dz7-38-39 5'-TTACAGTGGTATCGATTGGGACGTGTGGAGCGTCAGGATGGTATA-3'
Dz7-38-83 5'-TTACAGTGGTATCGATTGGGACGTGTGTAGCGTCAGGATGGGATCA-3'
Dz7-38-90 5'-TTACAGTGGTATCGATTGGGACGTGTGGAGCGTCAGGATGGGATCA-3'
Family II
Dz7-45-10 5'-AAGCAGCGGATGTGGTGTGACGCGGAGTCCCGAGTAGGGCTT-3'
DZ7-45-22 5'-AAGCAGCGGATGTGGTGTGACGCGGAGTCCCGGGTAGGGCTT-3'
Dz7-45-28 5'-AAGCAGCGGACGGATGGATGCGGAGTGCCTGAGTCAGGCCTT-3'

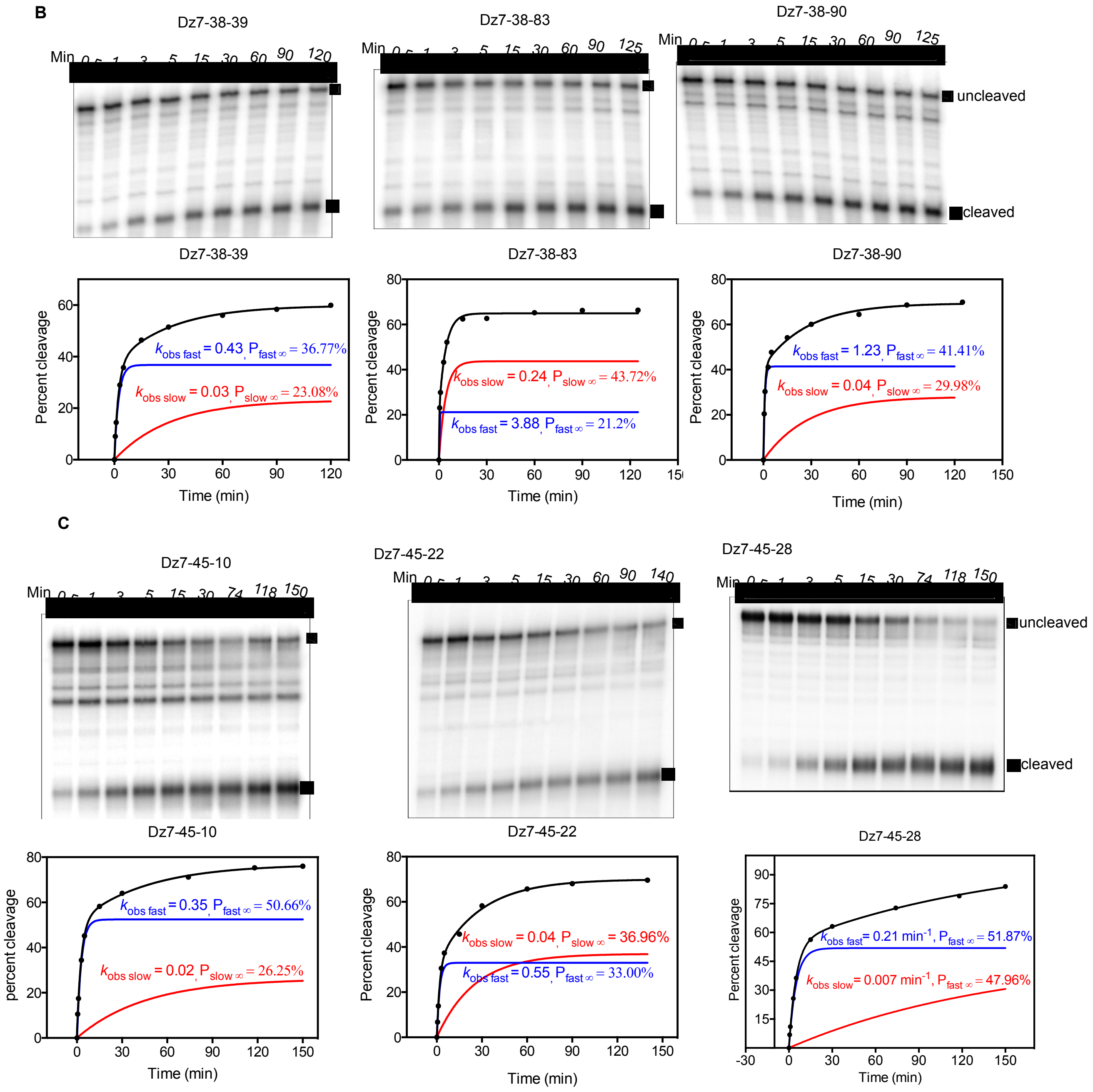

Figure S2. *Cis*-cleavage activity of DNAzymes.

Testing the need for modifications. 5X nucleotides cocktails containing natural counterpart(s) in replacement of any one or two, or all the three modifications (dA^{im}TP, dU^{ga}TP, dC^{aa}TP) contained in the 5X modified nucleotides cocktails were used into template-directed primer extension reactions for transcribing 15 pmole of partially modified or totally unmodified Dz7-38-32. Then kinetic *cis*-cleavage reactions were carried out following the same procedures described above in "**Kinetic analysis *in cis***". Time points were taken at 1, 5, 15, 60, 120, and 840 min for all different versions of Dz7-38-32, respectively. All samples were denatured by incubating at 95°C for 5 min, followed by snap-cooling on ice before resolving the supernatant after magnetization on denaturing PAGE (7%). Gel pictures were taken using Typhoon 9200 PhosphorImager (**Figure S3**).

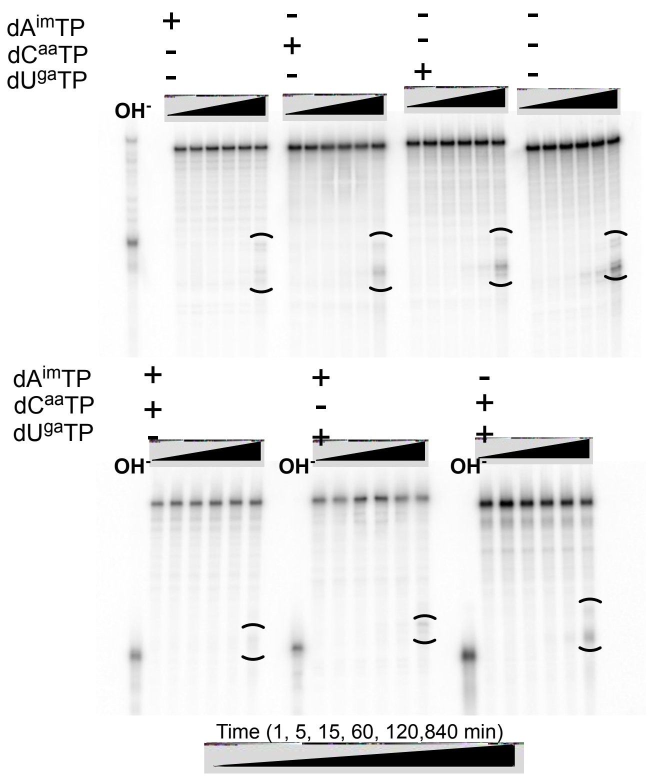

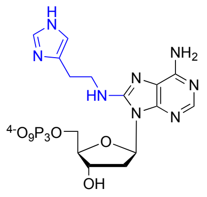

1 8-histaminyl-dATP (dA^{im}TP)

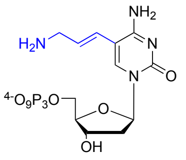

2 5-aminoallyl-dCTP (dC^{aa}TP)

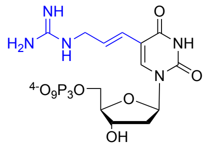

3 5-guanidinoallyl-dUTP (dU^{ga}TP)

Figure S3. The necessity of individual modifications for catalytic competence of Dz7-38-32. Left panel: gel images (7% PAGE) demonstrating the necessity of all three modifications for self-cleavage activity of Dz7-38-32. As a control, the elongated strands were also cleaved with 0.1 M NaOH at 65°C for 20 min to identify the migration of the cleavage products, as denoted with OH$^-$. Time points were 1, 5, 60, 120, and 840 min. Right panel: chemical structures of dA^{im}TP (**1**), dC^{aa}TP (**2**), and dU^{ga}TP (**3**).

Cleavage activity *in trans*.

Enzymatic synthesis of Dz7-38-32t. According to the methodology used for producing Dz10-66t,[4] 60 pmole of primer for transcribing of Dz7-38-32t (**ON 15**) was annealed to 60 pmol of the synthetic template of Dz7-38-32t containing a 5'-biotin moiety (**ON 14**) in a mixture of 55 μL solution containing 16 μL of 5X Sequenase buffer following the same protocol as described for the transcription of *cis*-cleaving Dz7-38-32. Following that, the catalytic strand was enzymatically synthesized at 32°C for 4 h using 26 units of Sequenase v2.0 (2 μl) in a final volume of 80 μL reaction supplemented with 16 μL of 5X modified nucleotides cocktail, 4 μL of DTT (100 mM), 2 μL of pyrophosphatase, and 1 μL of α-^{32}P-dGTP (~10 μCi). 4 μL of 0.5 M EDTA (pH 8.0) was added to a final concentration of 25 mM to quench the reaction at the end. 1 μL aliquot of this solution was diluted sequentially into 3X, 10X, 100X, 1000X, and 2000X solutions before the rest of the crude reaction was subjected to DNAzyme "workup".

The catalytic stand in duplex with its template was immobilized on prewashed streptavidin magnetic beads through the 5'-biotin moiety on the template. After two washes with 100 μL of wash buffer, the catalytic strand was stripped off with 30 μL of 0.1 M NaOH followed by immediate neutralization with 0.1 M HCl to obtain a final pH of ~7.5. The resulting DNAzyme solution was spun down through G25 column to desalt. 1 μL of the desalted DNAzyme was diluted for 10 times in H_2O, 1 μL of which along with 1 μL of each of the solutions resulting from sequential dilutions of the crude reaction were spotted on a TLC-plate (**Figure S4A**). An autoradiographic calibration curve (**Figure S4B**) represented by Equation 3.5 which relates autoradiographic density to signal volume and ultimately to the number of pmole α-^{32}P-dGTP (specificity) was generated by plotting logarithm of the signal intensities obtained from sequential dilutions against the logarithm of the dilution factors using linear regression. Then the concentration of DNAzyme produced can be calculated using **Equations S2.1** and **S2.2**.

Equation S2.1: $y = ax + b$

Equation S2.2: $$[DNAzyme] = \frac{\left[\alpha - {}^{32}P - dGTP\right] \times 10}{21 \cdot e^{-x}}$$

In **Equation S2.1,** a is the slope and b is the intercept of the linear calibration curve; in **Equation S2.2**, x is the calculated value after applying the logarithm of the signal intensity of the pure DNAzyme (**Figure 3.16A,** the spot in the blue rectangular) into **Equation 2.1**; the factors of 10 and 21 arise from the 10-fold dilution of the pure DNAzyme spotted on the TLC plate and the presence of 21 dGs in the polymerized sequence region of Dz7-38-32t, respectively.

A

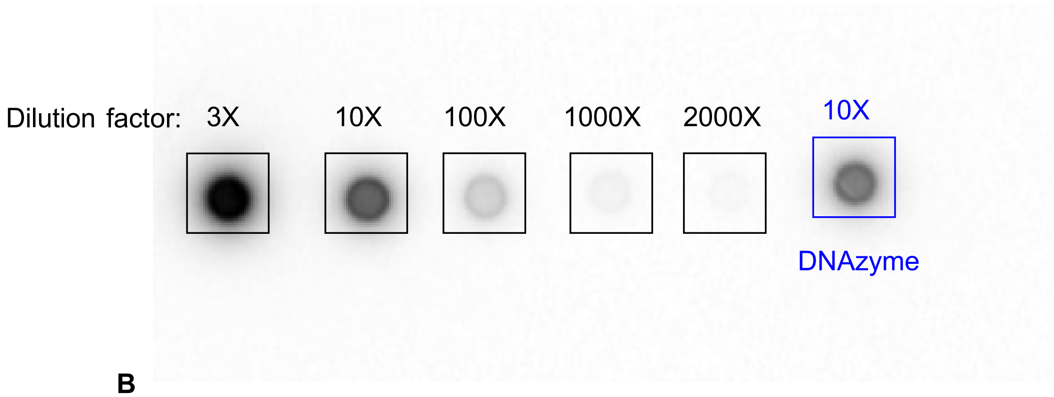

B

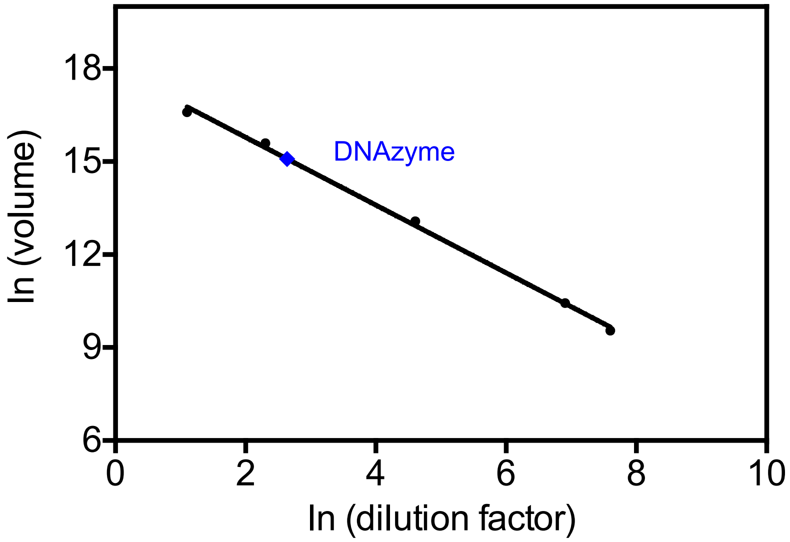

Figure S4. Concentration determination of Dz7-38-32t using autoradiographic calibration curve. (**A**) Autoradiogram of the standard curve representing the quantity of the total radioisotope at various dilutions. (**B**) Calibration curve of the logarithm of the signal intensity versus the logarithm of the dilution factor and the corresponding linear least square fit ($y = -1.093 \cdot x + 17.97$, $R^2=0.9978$). Dz7-38-32t, indicated by the blue square on the generated curve, has a concentration of 270 nM for this assay.

General protocol for *trans*-cleavage reactions. All the *trans*-reaction assays in this work were performed under multiple-turnover conditions. Normally 5 nM to 10 nM of DNAzyme ([Dz]) was used, and the concentrations of 5'-^{32}P-labeled substrate ([S]) used were always at least 10-fold in excess over that of the enzyme. DNAzyme and substrate were firstly incubated in 18 µL of solution at 95°C for 2 min followed by cooling on ice for 5 min before 2 µL of 10X *trans*-reaction buffer was added to initiate the cleavage reaction. All reactions were carried out in vessels coated with PAM oil to prevent material aggregation. Mineral oil was used to cover the reaction to minimize the effect of evaporation and condensation. At certain time points, 1.5 µL of sample was removed from reaction and added into 10 µL of gel loading buffer. Reaction products were separated by denaturing 20% PAGE, then visualized and quantified using PhosphorImager. Values of v_{obs} were calculated by fitting a minimum of five data points obtained over the first 10%-20% of cleavage reaction to linear least square regression.

Characterization of k_{cat} and K_M with the optimal 19 nt all-RNA (ON 17). These assays were carried out under multiple-turnover conditions in 1X *trans*-cleavage buffer containing 0.5 mM $MgCl_2$, and 150 mM KCl at both 30°C and 37°C. For assays at 30°C, the DNAzyme concentration was 5 nM, and substrate concentrations were 50, 100, 300, 500, 1000, 2000, and 4000 nM, respectively. For reactions at 37°C, the DNAzyme was used at 10 nM, and substrate concentrations were 100, 300, 500, 1000, 2000, 4000, and 8000 nM, respectively. Values of k_{cat} and K_M were determined by fitting obtained values of v_{obs} and corresponding substrate concentrations to Michaelis-Menten equation.

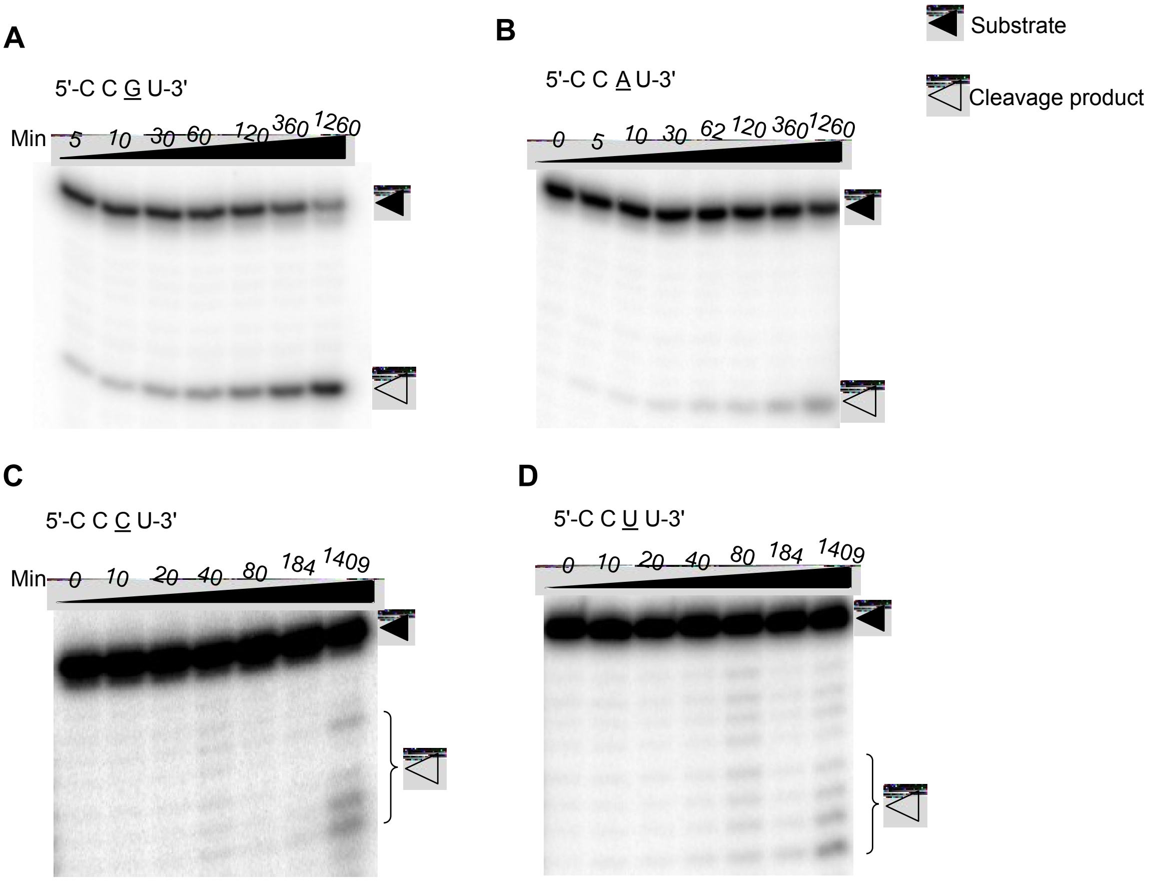

Figure S5. PAGE gel (20%) pictures for substrates containing different RNA bases at the cleavage site. (**A**) The original substrate containing rG at the cleavage site. (**B**) The substrate containing rA at the cleavage site. (**C**) The substrate containing rC at the cleavage site. (**D**) The substrate containing rU at the cleavage site. All the cleavage reactions were carried out under simulated physiological ionic conditions: 50 mM cacodylate (pH 7.45), 0.5 mM Mg^{2+}, 150 mM KCl, at 30°C. [Dz]=5 nM, [S]=1 μM.

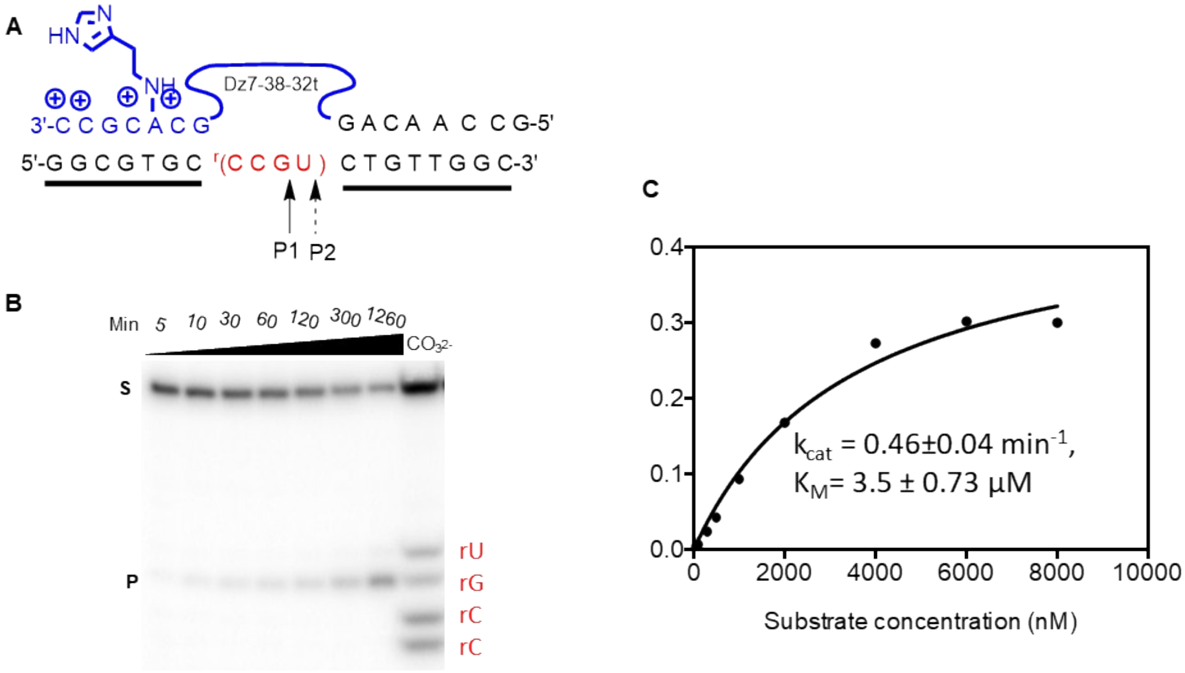

Figure S6. Activity of Dz7-38-32t on the chimeric DNA/RNA substrate. (A) Dz7-38-32t in complex with the chimeric DNA/RNA substrate containing DNA bases in the substrate-recognition arms (underlined) and unpaired RNA bases (in red) at the cleavage region. (B) Representative PAGE gel (20%) of *trans*-cleavage reaction in the presence of 1 μM substrate. S: the intact substrate; P: cleavage product. Reaction conditions: 50 mM sodium cacodylate (pH 7.45), 0.5 mM $MgCl_2$, 150 mM KCl, at 30°C. [Dz]=5 nM, [S]=1 μM. (C) Multiple-turnover profile. Substrate concentrations are 50, 100, 300, 500, 1000, 2000, 4000, 6000, and 8000 nM. k_{cat}=0.46±0.04 min^{-1}, K_M=3.5 ± 0.73 μM (R^2=0.99).

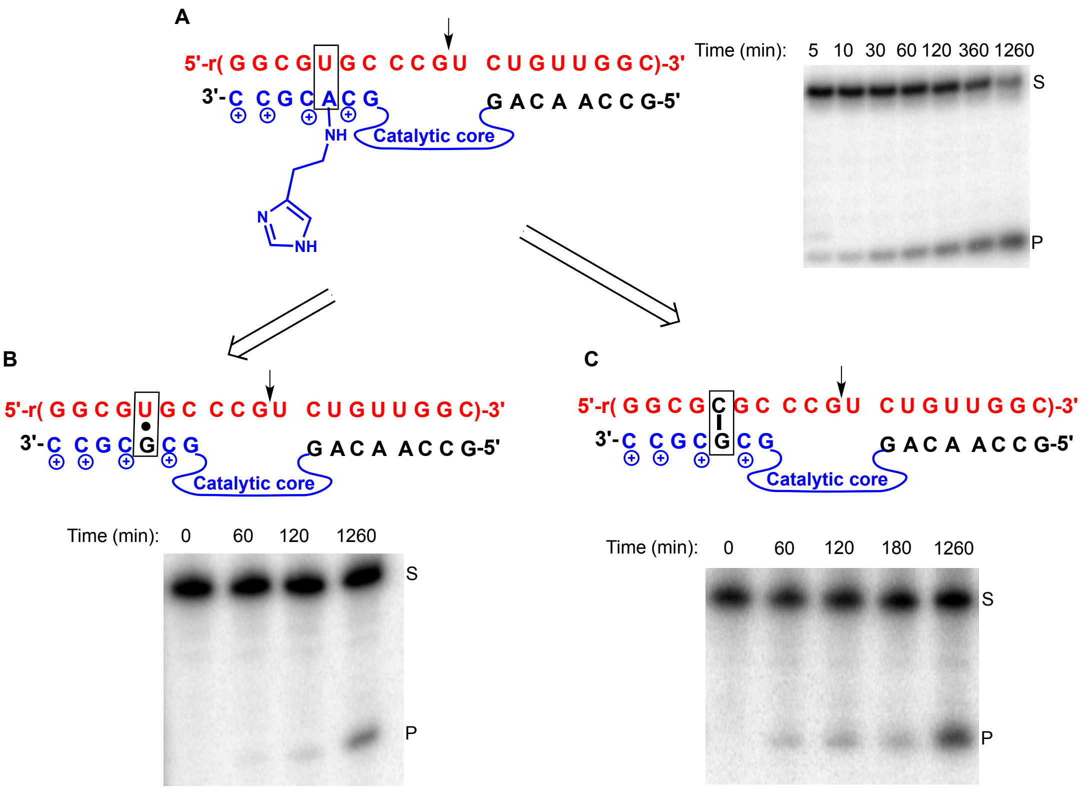

Figure S7. The effect of changing the dA^{im} in the modified 3' substrate-recognition arm for dG: S: the intact 19 nt all-RNA substrate; P: 5' cleavage product. Arrow indicates the site of cleavage. (A) Dz7-38-32t targeting the original 19 nt all-RNA substrate. (B) "dA^{im} to dG" mutated Dz7-38-32t targeting the original 19 nt all-RNA substrate. (C) "dA^{im} to dG" mutated Dz7-38-32t targeting the all-complementary RNA substrate. Reaction conditions: 50 mM cacodylate (pH 7.45), 0.5 mM Mg^{2+}, 150 mM KCl, at 30°C. [Dz]=5 nM, [S]=1 μM.

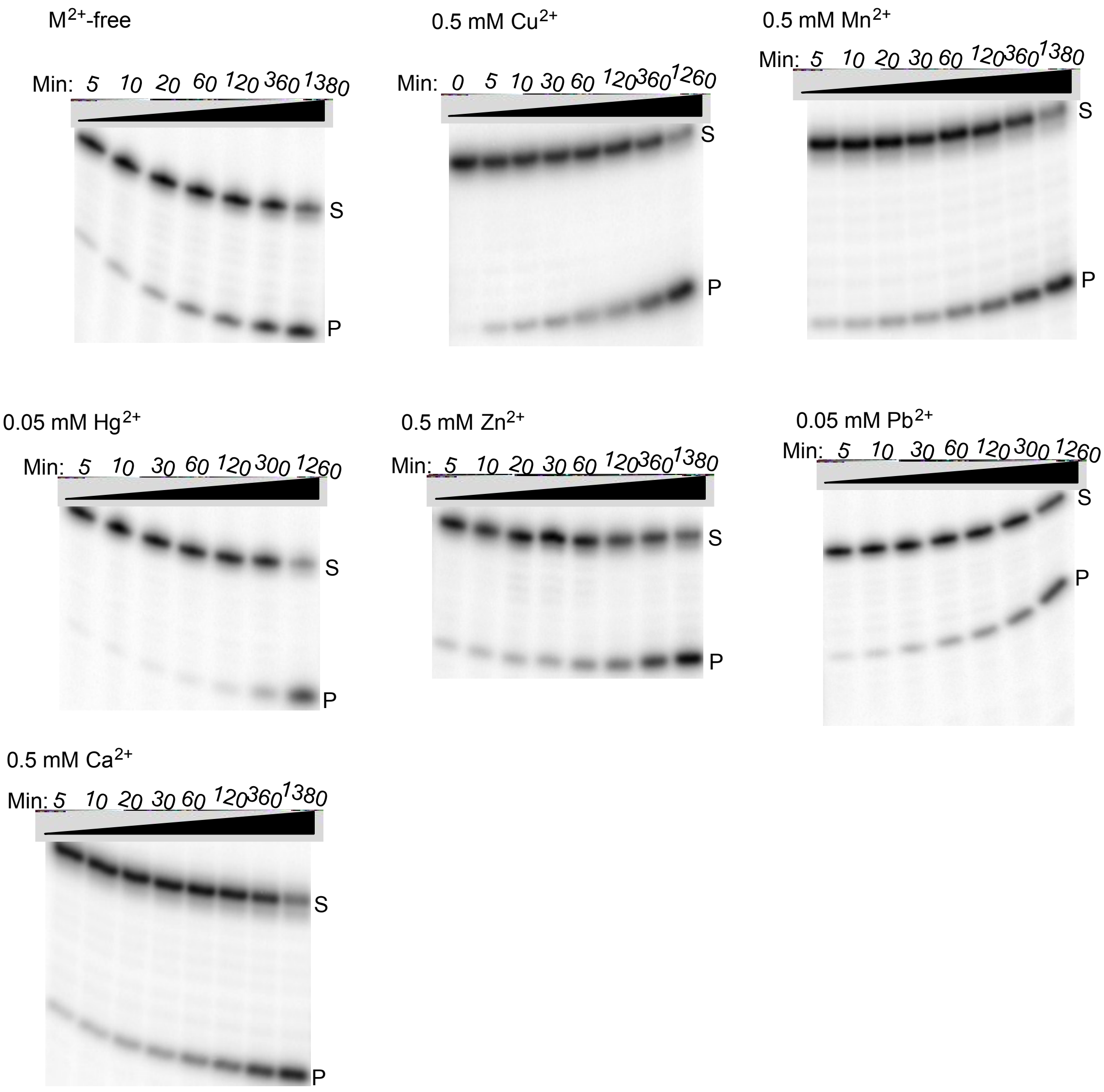

Figure S8. Effects of divalent metal ions on the *trans*-cleavage activity of Dz7-38-32t. S: the intact substrate; P: the cleavage product. All the reactions were carried out in 50 mM cacodylate buffer (pH 7.45) containing 150 mM KCl, and the tested concentration of M^{2+}, at 30°C. P: cleavage product. [Dz]=5 nM, [S]= 1 μM.

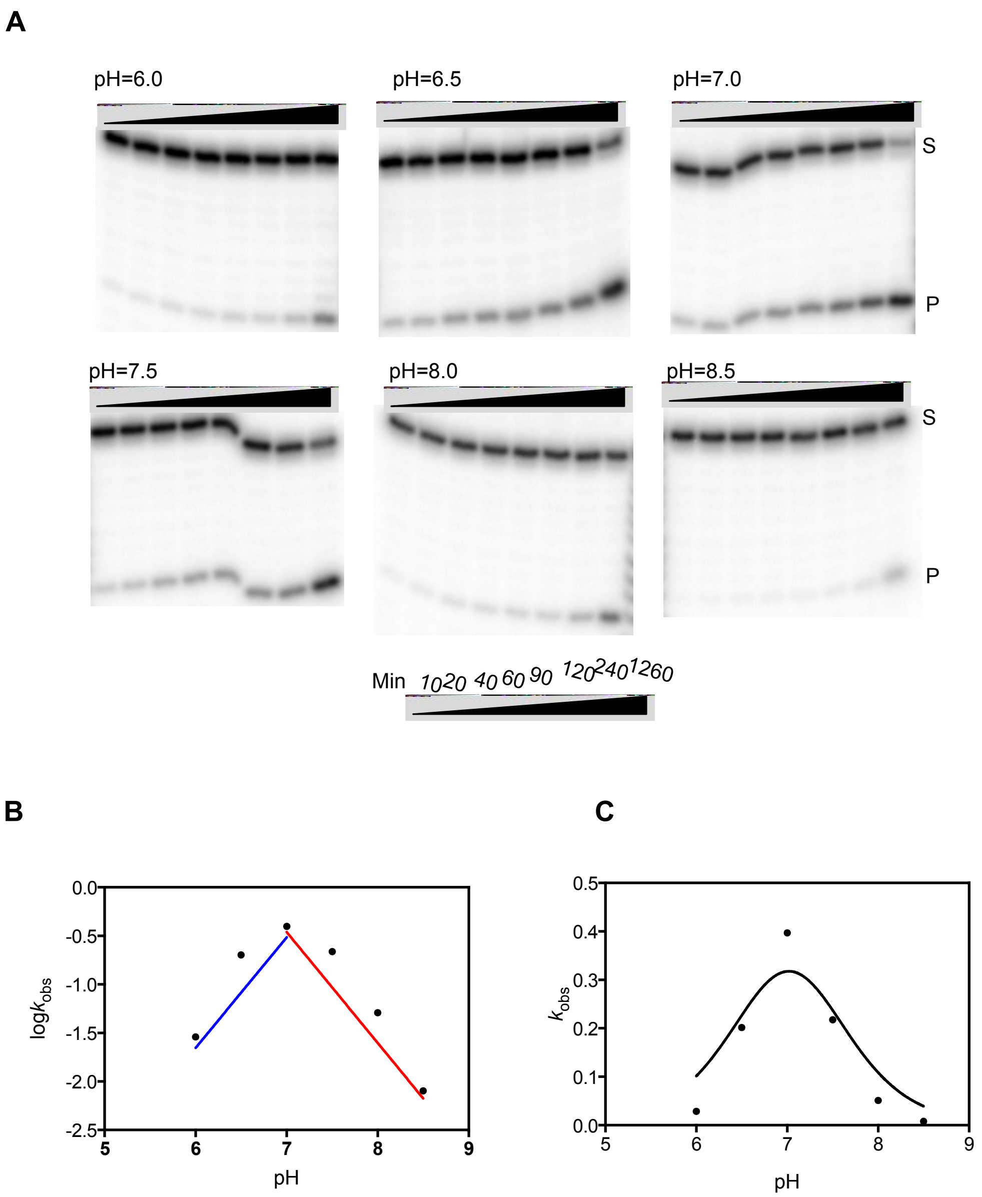

Figure S9. The pH dependence study of Dz7-38-32t. (**A**) Gel (20%) pictures of *trans*-cleavage reactions under 1X pH variant buffers containing 0.5 mM $MgCl_2$, 150 mM KCl, at 30°C. S: the intact substrate, P: cleavage product. pH 6.0-7.0 buffers: 50 mM sodium cacodylate. pH 7.5-8.5 buffers: 50 mM sodium phosphate. [Dz]=7.5 nM, [S]=1.5 μM. (**B**) The logk_{obs}-pH linear regressions in pH 6-7 (blue line, y=1.139X-8.284), and pH 7-8.5 (red line, y=-1.143X+7.745), respectively. (**C**) pH-rate profile generated by fitting values of k_{obs} and pH to **Equation S3**:

$$k_{obs} = \frac{k_{max}}{1 + 10^{\left(pH - pK_a^1\right)} + 10^{\left(pK_a^2 - pH\right)} + 10^{\left(pK_a^2 - pK_a^1\right)}}$$

, where pK_a^1 and pK_a^2 are the acid dissociation constants of two catalytically relevant groups in a two-step protonation-deprotonation mechanism.

Reference

1. Lam, C.; Hipolito, C.; Perrin, D. M., Synthesis and enzymatic incorporation of modified deoxyadenosine triphosphates. *European Journal of Organic Chemistry* 2008, *2008* (29), 4915-4923.
2. Hollenstein, M.; Hipolito, C. J.; Lam, C. H.; Perrin, D. M., A self-cleaving DNA enzyme modified with amines, guanidines and imidazoles operates independently of divalent metal cations (M2+). *Nucleic Acids Res* 2009, *37* (5), 1638-49.
3. Cadwell, R.; Joyce, G., Mutagenic PCR. *PCR methods and applications* 1994, *3* (6), S136.
4. Hollenstein, M.; Hipolito, C. J.; Lam, C. H.; Perrin, D. M., A DNAzyme with three protein-like functional groups: enhancing catalytic efficiency of M2+-independent RNA cleavage. *Chembiochem : a European journal of chemical biology* 2009, *10* (12), 1988-92.
